# Bioengineering an improved three-dimensional vascularized co-culture model for studying Neuron–Microglia interactions

**DOI:** 10.1016/j.bioactmat.2025.09.008

**Published:** 2025-09-10

**Authors:** Yinhe Han, Lina Guo, Mingqi Wang, Zhen Cao, Xu Zheng, Xinyu Wang, Lingling Jin, Xiaoqing Wei, Xiuli Wang, Jie Zhao

**Affiliations:** a College of Basic Medical Science, Dalian Medical University, Dalian, 116044, China; b National-Local Joint Engineering Research Center for Drug-Research and Development (R&D) of Neurodegenerative Diseases, Dalian Medical University, Dalian, 116044, China

**Keywords:** Human-induced neural stem cells (hiNSCs), Microglia, Human vascular organoids, Vascularization, Immune-neurovascular, SDF-1/CXCR4

## Abstract

Interactions among neurons, microglia, and endothelial cells (ECs) —the principal components of the neurovascular unit (NVU)—are vital for maintaining central nervous system (CNS) homeostasis and are implicated in numerous neurological disorders. However, mechanistic insights into their crosstalk remain limited due to the lack of physiologically relevant *in vitro* models. In this study, we present an improved 3D vascularized tri-culture model that integrates human-induced neural stem cells (hiNSCs), human vascular organoids (hVOs), and microglia within a geometrically engineered silk fibroin scaffold. This platform effectively recapitulates critical features of the native CNS microenvironment, including spatial neurovascular patterning and cell-type-specific interactions. Within this model, hVOs significantly promoted neuronal differentiation of hiNSCs, resulting in extended axonal networks and improved neurovascular alignment. Microglial effects were found to be phenotype-dependent: both resting (M0) and pro-inflammatory (M1) microglia inhibited hiNSCs differentiation and vascular development, with M1 cells exerting the strongest suppressive influence. In contrast, anti-inflammatory (M2) microglia displayed the least inhibitory effect and even modestly supported neurovascular maturation. Mechanistic studies revealed that M2 microglia cooperate with hVOs via the stromal cell-derived factor 1 (SDF-1)/C-X-C chemokine receptor type 4 (CXCR4) signaling axis to promote neuronal differentiation. To our knowledge, this represents the first demonstration of SDF-1/CXCR4-mediated immune-neurovascular interaction within a human tri-culture system. Thereafter, this 3D vascularized co-culture model provides a physiologically relevant *in vitro* platform to investigate neuroimmune and neurovascular interactions. It holds broad potential for mechanistic studies in neurodevelopment and neurodegeneration, drug evaluation, and the development of regenerative therapies.

## Highlights

• Oriented silk fibers scaffold enhance axon elongation and neurovascular organization.
• Resting and M1 microglia suppress neural and vascular differentiation *in vitro*.
• HVOs promote axon outgrowth; M2 microglia support hiNSCs neural differentiation.
• M2 microglia and hVOs promote hiNSCs neurogenesis via SDF-1/CXCR4 signaling.

## Graphical abstract

**Figure:**
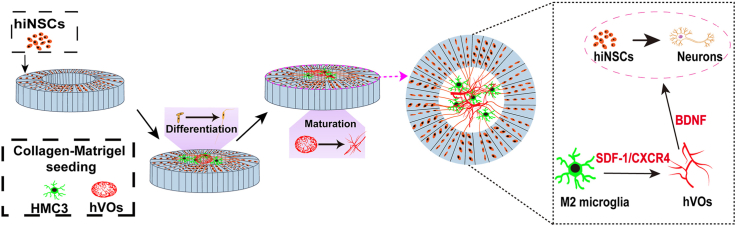

## 1 Introduction

The central nervous system (CNS) relies on the precise and dynamic interplay among various cell types, with neurons, endothelial cells (ECs), and microglia forming the functional core of the immune-neurovascular microenvironment [1,2]. These cell types work orchestrally to maintain neural homeostasis and regulate essential physiological processes such as cerebral blood flow, synaptic plasticity, immune surveillance, and metabolic support.

Neurons depend on the vascular network not only for nutrient and oxygen delivery but also for modulating activity-dependent vascular responses [3,4]. Endothelial cells, which line the blood-brain barrier (BBB), contribute to the maintenance of CNS homeostasis by controlling molecular transport, releasing neurotrophic factors, and mediating inflammatory signaling [5,6]. Microglia, the resident immune cells of the CNS, serve critical roles in neurodevelopment, synaptic remodeling, and response to injury and disease [7]. In particular, microglia exhibit remarkable phenotypic plasticity, adopting context-dependent functional states. The pro-inflammatory M1 phenotype is characterized by the release of cytokines, such as TNF-α and IL-1β, which often result in neurotoxicity and vascular compromise. In contrast, the anti-inflammatory M2 phenotype secretes trophic factors that support angiogenesis, neurogenesis, and tissue repair [8,9]. The nature of microglial interactions with neurons and ECs, and how these interactions influence cellular fate and function under physiological and pathological conditions, remains incompletely understood.

A major barrier to elucidating these mechanisms lies in the lack of physiologically relevant and tractable *in vitro* models capable of recapitulating the tri-cellular architecture and signaling dynamics of the immune-neurovascular microenvironment. Traditional two-dimensional (2D) co-culture systems and transwell-based BBB models fail to reproduce the three-dimensional (3D) spatial organization and cellular complexity of brain tissue. Brain organoids, due to their self-organizing properties, have emerged as valuable tools for studying human neurodevelopment and disease [10]. However, conventional organoid models often lack vascularization and immune components, leading to limitations in nutrient diffusion, cellular maturation, and accurate modeling of immune-neurovascular interactions [11].

Other efforts have explored microfluidic brain-on-a-chip platforms that allow spatiotemporal control and the application of fluidic shear stress. While promising, these systems are often limited by low cell throughput, operational complexity, and challenges in fully integrating all relevant components of the immune–neurovascular microenvironment [12]. 3D bioprinting technologies allow programmable spatial patterning, yet challenges remain in achieving sufficient resolution, biocompatibility, and multi-lineage cell integration [13]. *In vivo* transplantation of human-derived organoids into immunodeficient mice has enabled studies of cellular integration and vascularization [14,15], however, these approaches are confounded by interspecies differences and the complexity of the host microenvironment, which makes it difficult to isolate human-specific cell-cell interactions.

To address these limitations, we developed a structurally defined, spatially organized, and analytically tractable 3D human tri-culture model—drawing inspiration from the brain-mimetic architecture pioneered by Kaplan DL. et al. This system integrates human-induced neural stem cells (hiNSCs), human vascular organoids (hVOs), and microglia within geometrically engineered silk fibroin (SF) scaffolds designed to support directed neuronal outgrowth. hiNSCs were seeded onto the oriented lamellar surface of the scaffold, where they differentiated into neurons and extended axons toward a central cavity populated by hVOs and human microglial clone 3 (HMC3) cells. This spatial configuration enables the organized assembly of neurovascular and neuroimmune components within a biomimetic microenvironment, facilitating both the recapitulation of *in vivo*-like phenotypes and the mechanistic interrogation of intercellular interactions.

Using this model, we found that hVOs markedly enhanced neuronal differentiation and axonal outgrowth. Moreover, the effects of microglia were phenotype-dependent. Further investigation identified the SDF-1/CXCR4 signaling axis as a key mediator in this tri-cellular interaction network—a pathway that had not been previously characterized in this context. This platform opens new avenues for investigating the cellular and molecular mechanisms underlying neuroimmune and neurovascular regulation, and holds significant promise for disease modeling, drug screening, and regenerative medicine applications.

## 2 Materials and methods

### 2.1 Cell culture

hiNSCs (ScienCell, CA, USA) were maintained and expanded in mTeSR™ Plus Basal Medium (STEMCELL Technologies, Vancouver, Canada) supplemented with 5 × Supplement (STEMCELL Technologies) and cultured on Matrigel (Corning, ME, USA)-coated 6-well plates. To induce neural differentiation, hiNSCs were cultured in a differentiation medium composed of Neurobasal medium (Gibco, CA, USA) supplemented with 2 % B27(Gibco), 1 % Glutamax (Gibco), and 1 % Penicillin-Streptomycin Solution (P/S, Solarbio Science & Technology, Beijing, China). To ensure quality control, only cells within passages 3–6 were used for all the experiments in this study.

Human embryonic stem cells (hESCs), line H9 (WiCell, WI, USA), were maintained in mTeSR™ Plus Basal Medium (STEMCELL Technologies) supplemented with 5 × Supplement (STEMCELL Technologies) and cultured on Matrigel (Corning)-coated 6-well plates. Cells were subcultured when they reached 70–80 % confluence, and the medium was replaced every two days. To preserve the undifferentiated state of the cells, colonies showing differentiated morphology were carefully removed using a glass pipette under phase-contrast microscopy (Zeiss, Wetzlar, Germany).

Human microglial cell line (HMC3, ATCC) was cultured in Minimum Essential Medium (MEM, Gibco) supplemented with 10 % fetal bovine serum (FBS, ExCell, Australia) and 1 % P/S (Solarbio), and maintained at 37 °C with 5 % CO_2_ in a humidified incubator. To induce distinct polarization states, HMC3 cells were stimulated with 2 μg/mL lipopolysaccharide (LPS, Sigma, St. Louis, MO, USA) for M1 polarization or with 60 ng/mL interleukin-4 (IL-4, MCE, New Jersey, USA) for M2 polarization. After 24 h of stimulation, the polarized cells were collected and used for subsequent co-culture experiments. Unstimulated HMC3 cells (M0) were used as resting microglia and served as the control group.

### 2.2 hVOs preparation

hVOs were generated based on previously reported protocols [16]. Firstly, H9 cells were cultured under suspension conditions to form embryoid bodies (EBs). To initiate mesodermal differentiation, EBs were transferred into an induction medium comprising equal volumes of Neurobasal and DMEM/F12 medium, supplemented with 0.5 % Glutamax, 2 % B27, 1 % N2 supplement, 1 % P/S (all from Gibco), 0.143 mM β-mercaptoethanol (Sigma), 30 ng/mL BMP4 (Proteintech, Wuhan, China), and 12 μM CHIR99021 (APExBIO, Houston, TX, USA). After 3 days, mesodermal induction was followed by vascular lineage specification using a medium composed of equal parts Neurobasal and DMEM/F12 medium, 0.5 % Glutamax, 2 % B27, 1 % N2 supplement, 1 % P/S, 0.143 mM β-mercaptoethanol (Sigma), 100 ng/mL VEGFA (Proteintech), and 2 μM Forskolin (Sigma). On day 7, the medium was changed to StemPro®-34 SFM medium (vascular induction medium) (Gibco) supplemented with 1 % Glutamax, 1 % P/S, 15 % FBS, 100 ng/mL bFGF (Peprotech, New Jersey, USA), and 100 ng/mL VEGFA (Proteintech). Organoids were embedded in a collagen/Matrigel composite hydrogel (3:1, v/v) and cultured in vascular induction medium, with medium changes every three days. After five days, mature hVOs were obtained for characterization and downstream applications. All hVOs were harvested on day 12 of directed differentiation, a time point selected based on preliminary data demonstrating consistent structural and functional maturation. Imaging was performed using a confocal laser scanning microscope (CLSM; Leica TCS SP8, Wetzlar, Germany).

### 2.3 Silk fibroin solution preparation and oriented scaffolds fabrication

The SF aqueous stock solution was generously provided by Soochow University (Suzhou, China). In brief, SF fibers were extracted from Bombyx mori silkworms (China) and degummed by boiling in a 0.02 M Na_2_CO_3_ (Solarbio) solution to remove sericin. The purified fibroin was then dissolved in 9.3 M lithium bromide (LiBr, Aladdin, Shanghai, China) at 60 °C for 4 h. Following dissolution, the solution was subjected to dialysis for 4–5 days to remove residual salts, then centrifuged to remove insoluble particles. The final concentration of the SF solution was determined and used for subsequent experiments.

Oriented SF scaffolds were prepared with minor modifications to a reported protocol [17]. SF solutions (6 %, 5 %, 4 %) were dispensed into 15 mL tubes, frozen upright in liquid nitrogen for 30 min, lyophilized for 96 h, and autoclaved to induce β-sheet formation. The resulting donut-shaped scaffolds measured 1–2 mm in thickness, with outer and inner diameters of 6 mm and 2 mm, respectively.

Porous SF scaffolds were fabricated using a salt-leaching method established by Kaplan's group [18,19]. A 5 % SF solution was processed and cast with porogens to generate interconnected pores (500–650 nm). Donut shapes were created by punching 2 mm central holes in 6 mm SF disks.

### 2.4 Performance testing of oriented SF scaffolds

#### 2.4.1 Swelling test

The scaffold was immersed in ultra-pure water at room temperature for 24 h. After excess water was removed, the wet weight (Ws) of the scaffold was recorded. The sample was then dried overnight in a vacuum oven at 60 °C, and the dry weight (Wd) was measured. This procedure was repeated three times for each group. Three replicates were performed for each group. The swelling ratio of the scaffold was calculated using the formula: ((Ws - Wd)/Wd) × 100 %.

#### 2.4.2 Mechanical properties

Mechanical strength was evaluated using cylindrical scaffolds (8 mm in diameter and 5 mm in height), which were soaked in ultrapure water for 12 h at room temperature. The compression properties were measured at room temperature using an Instron 3365 testing machine (Instron, Norwood, MA, USA) equipped with a 100 N load cell. A compressive force was applied until the scaffold was compressed to 80 % of its original height, after which the load was released. The compressive modulus was determined from the linear elastic region of the stress-strain curve. Data were reported as the average of 4–6 tests, each involving three parallel scaffolds.

#### 2.4.3 Fourier transform infrared spectroscopy (FTIR)

The secondary structures of the scaffolds were analyzed using a Nicolet FTIR 5700 spectrometer (Thermo Scientific, FL, USA) in attenuated total reflection (ATR) mode. A thin layer of SF scaffold (2 mm in thickness) was cut and subjected to FTIR analysis. Each measurement involved 64 scans with a resolution of 4 cm^−1^ and a wave number range of 400–4000 cm^−1^. Each group was analyzed in triplicate.

### 2.5 Construction of SF scaffold-based 3D cultures

The SF scaffolds were pre-coated with 0.5 mg/mL type I rat tail collagen (Corning) to enhance cell adhesion. Dissociated hiNSCs were seeded onto each scaffold at a density of 1 × 10^6^ cells per scaffold. The cell-loaded scaffolds were carefully transferred into 48-well plates containing neural differentiation medium and incubated overnight to promote initial cell attachment. For the mono-culture of hiNSCs, after 2 days of cell seeding, each hiNSCs-loaded scaffold was filled with 20 μL of the Collagen/Matrigel composite hydrogel, and incubated for 2 h at 37 °C to allow complete gelation. The scaffolds were then maintained in neural differentiation medium, with medium changes every 3 days. For the construction of the co-culture and tri-culture model, 15 days after initial hiNSCs seeding, either a single hVO, 1 × 10^5^ HMC3 cells (M0, M1, or M2 phenotype), or both were resuspended in 20 μL of collagen/Matrigel hydrogel and added to the central region of the scaffold. After gelation, the scaffolds were transferred to 48-well plates and cultured in co-culture medium optimized to support the survival and function of all involved cell types. Mono-culture of hiNSCs (without hVOs and HMC3 cells) served as controls under identical conditions and seeding density. Samples were harvested 5 days after the initiation of co-culture or tri-culture.

### 2.6 Electron microscopy

Cell-loaded scaffolds were fixed in 2.5 % glutaraldehyde solution (Solarbio) at 4 °C overnight. After thorough rinsing with phosphate-buffered saline (PBS, Solarbio), the samples were dehydrated through a graded ethanol series and subsequently lyophilized. The dried specimens were sputter-coated with a thin layer of gold and mounted onto aluminum stubs for scanning electron microscopy (SEM) imaging. Surface morphology was examined using a J JSM-IT300 scanning electron microscope (SEM, JEOL, Japan).

For transmission electron microscopy (TEM) analysis, samples were post-fixed, embedded in Epon 812 resin, and sectioned into ultrathin slices. TEM imaging was performed using a JEM-2000EX transmission electron microscope (TEM, JEOL, Japan).

### 2.7 Cell viability staining

Cell viability was evaluated using a Calcein-AM/EthD-1 staining kit (Invitrogen) following the manufacturer's instructions. After harvesting and rinsing the 3D cultures three times with PBS, the samples were incubated in the Calcein-AM/EthD-1 working solution at 37 °C for 3 h. Following incubation, the samples were washed with PBS and transferred to glass-bottom Petri dishes. Fluorescent images were acquired using Leica TCS SP8 CLSM.

### 2.8 Histological staining

#### 2.8.1 Hemotoxylin & Eosin (H&E) staining

Samples were fixed in 4 % paraformaldehyde (Solarbio) at 4 °C overnight for paraffin-embedded sample preparation. Paraffin sections (5 μm) were prepared by the Department of Pathology, Dalian Medical University (Dalian, China). Sections were deparaffinized, rehydrated through a graded ethanol series, and subjected to H&E staining according to standard protocols [20]. Images were captured under a fluorescence microscope imaging system (BZ-X800, Keyence, Osaka, Japan).

#### 2.8.2 Immunofluorescence staining

The samples were fixed overnight in 4 % paraformaldehyde, washed with PBS, and then permeabilized and blocked with 0.5 % Triton X-100 and 3 % BSA for 2 h at room temperature. The following primary antibodies were applied overnight at 4 °C: Rabbit anti-human CD11b (1:200, Wanleibio, Shengyang, China), Rabbit anti-human CD163 (1:200, Wanleibio), Alexa Fluor® 647 mouse anti-human TUJ1 (1:200, BD biosciences, Franklin Lakes, NJ, USA), Alexa Fluor® 647 mouse anti-human CD31 (1:200, BD biosciences), Rabbit anti-human CD31 (1:200, Invitrogen, Carlsbad, CA, USA), and Rabbit anti-human PDGF Receptor β (1:100, CST, Danvers, MA, USA), Rabbit anti-human αSMA (1:200, Abcam), Rabbit anti-human COL4 (1:200, Proteintech), Rabbit anti-human BDNF (1:100, Abcam, Cambridge, UK), Rabbit anti-human PSD95 (1:100, Proteintech), Mouse anti-human IBA1 (1:200, Abcam). After washing with PBS, the samples were incubated with Alexa Fluor® 488- or 568-conjugated goat anti-rabbit/mouse IgG (1:200, Invitrogen) for 2 h at room temperature. Nuclei were counterstained with DAPI (2 μg/mL, Research Organics, Ohio, USA). Images were captured using a Leica TCS SP8 CLSM (Germany).

#### 2.8.3 Image semi-quantification analysis

To quantitatively assess the morphological characteristics of differentiated neurons, images obtained from phase-contrast or immunofluorescence microscopy were preprocessed using ImageJ software (NIH, Bethesda, MD, USA). Neurite tracing was performed with the NeuronJ and Simple Neurite Tracer plugins [[21], [22], [23]]. At least five random fields were analyzed per group, and the results were averaged for semi-quantitative analysis.

For neurite counting, the total number of distinct TUJ1-positive neurite processes per field was manually counted. For neurite length analysis, individual neurites were manually traced using NeuronJ. Each neurite was measured from its point of origin to the distal tip and converted to micrometers based on the calibrated image scale. For each image field, the total neurite length was calculated, and average lengths were determined across multiple fields. Data analysis followed the methodology described by Popko et al. [22].

To assess neurite integrity, the Simple Neurite Tracer plugin was used to generate skeletonized tracings from which the number of neurite endpoints per field was automatically quantified. The neurite integrity index was calculated by dividing the total neurite length (μm) by the number of endpoints. A higher integrity index reflects longer, more continuous neurites with fewer interruptions, indicating improved structural preservation and connectivity.

### 2.9 Ac-LDL uptake in hVOs

To evaluate the endothelial functionality of hVOs in tri-culture, hVOs located in the central pores of the SF scaffolds were physically isolated into 1.5 mL tubes and rinsed once with PBS. Subsequently, hVOs from different groups were incubated with 10 μg/ml DiI-labeled acetylated low-density lipoprotein (DiI-ac-LDL, Invitrogen) for 2 h at 37 °C. Following incubation, nuclei were counterstained with Hoechst 33342 (1:1000, Invitrogen) for 30. After thorough washing with PBS, DiI-ac-LDL uptake by ECs in hVOs was visualized using SP8 CLSM.

### 2.10 Real-time quantitative PCR (RT-qPCR)

To enable region-specific analysis in the tri-culture system, we separately collected the SF scaffold region (enriched in neuronal cell bodies) and the central hydrogel region (containing hVOs and microglia). For neuron-related analyses, the central area was carefully excluded to avoid contamination. For hVOs-related analyses, central hydrogel plugs were collected after time-controlled enzymatic digestion (1 mg/mL collagenase type I, 5–8 min at 37 °C) and gentle mechanical separation, which preserved hVOs integrity while minimizing microglial contamination. After spatial separation of tissue regions, total RNA was extracted from each compartment to assess region-specific gene expression.

Total RNA was isolated from the samples using Trizol reagent (Ambion Life Technologies, Carlsbad, CA, USA) following the manufacturer's instructions. Subsequently, 1 μg of RNA was reverse-transcribed into cDNA using a synthesis kit from TaKaRa (Shiga, Japan). RT-PCR was performed using the Agilent Technologies System (China) with the SYBR® Premix Ex Taq™ II Kit (Takara). The thermal cycling protocol consisted of 40 cycles of denaturation at 95 °C for 3 s, annealing at 58 °C for 30 s, and extension at 72 °C for 30 s. Relative gene expression levels were calculated using the 2^−ΔΔCt method, with normalization to *GAPDH*. Each sample was analyzed in triplicate. Primer sequences are listed in Table S1.

### 2.11 Western blot assays

For protein analysis, the same region-specific sampling strategy described for RNA extraction was employed to separately isolate the neuronal-enriched SF scaffold region and the hVOs/microglia-containing central hydrogel region. Samples were collected at the designated time points and lysed using RIPA buffer (Servicebio, Wuhan, China) according to the manufacturer's protocol. Total protein concentration was determined using a BCA Protein Assay Kit (Seven, Beijing, China). Equal amounts of protein were separated by SDS-polyacrylamide gel electrophoresis (SDS-PAGE) and transferred onto polyvinylidene fluoride (PVDF) membranes (Millipore, Massachusetts, USA). Membranes were blocked with 5 % non-fat milk in TBST for 1.5 h at room temperature and then incubated overnight at 4 °C with the following primary antibodies: Rabbit anti-human Neurofilament Heavy Chain (1:1000, Abcam), Rabbit anti-human BDNF (1:1500, Abcam), Rabbit anti-human OCT4 (1:2000, Abcam), Rabbit anti-human CD86 (1:1000, Wanleibio), Rabbit anti-human CD163 (1:1000, Wanleibio), Rabbit anti-human SDF-1 (1:1000, Wanleibio), Rabbit anti-human PSD95 (1:1000, Wanleibio), Rabbit anti-human GAPDH (1:5000, Servicebio), and Mouse anti-human Beta-Actin (1:5000, Proteintech). After washing, membranes were incubated with peroxidase-conjugated secondary antibodies, Goat anti-Mouse IgG (H + L) (1:5000, Proteintech) and HRP-conjugated Goat anti-Rabbit IgG (1:5000, Abbkine, Wuhan, China), for 1.5 h at room temperature. Protein bands were visualized using enhanced chemiluminescence (ECL Plus, Seven) reagents and imaged using a gel documentation system. Band intensities were quantified using ImageJ software and normalized to β-actin or GAPDH as internal loading controls.

## 3 Statistical analysis

All data are presented as the mean ± standard deviation (SD) from at least three independent biological replicates. Statistical analyses were conducted using GraphPad Prism 8 (GraphPad Software, CA, USA). For comparisons between two groups, an unpaired two-tailed Student's t-test was applied. For analysis involving three or more groups, one-way ANOVA followed by Tukey's post hoc test was performed. Statistical significance was defined as ∗*P* < 0.05, ∗∗*P* < 0.01, and ∗∗∗*P* < 0.001.

## 4 Results

### 4.1 Characterization of oriented donut-shaped SF scaffolds

Donut-shaped SF scaffolds with concentrations of 4 %, 5 %, and 6 % were fabricated and subjected to physical characterization, compression testing, and β-sheet content analysis (Fig. S1A). The 4 % SF scaffolds exhibited insufficient mechanical strength, making them unsuitable for handling and long-term culture, whereas the 6 % scaffolds were excessively stiff with poor absorbability, which hindered nerve cell migration (Fig. S1B–E). To assess their impact on neural differentiation, hiNSCs were seeded onto scaffolds of different concentrations, and the expression of neural differentiation-related genes was analyzed. The 5 % scaffolds demonstrated the highest expression of the neuronal marker nuclear factor of T-cells *(NFAT)* and the lowest expression of the stemness marker SRY-box transcription factor 2 (*SOX2)* (Fig. S1F), indicating their superior support for neuronal differentiation. Based on the optimal balance of mechanical properties and biological effects, the 5 % SF scaffolds were selected for subsequent experiments.

**Fig. 1:**
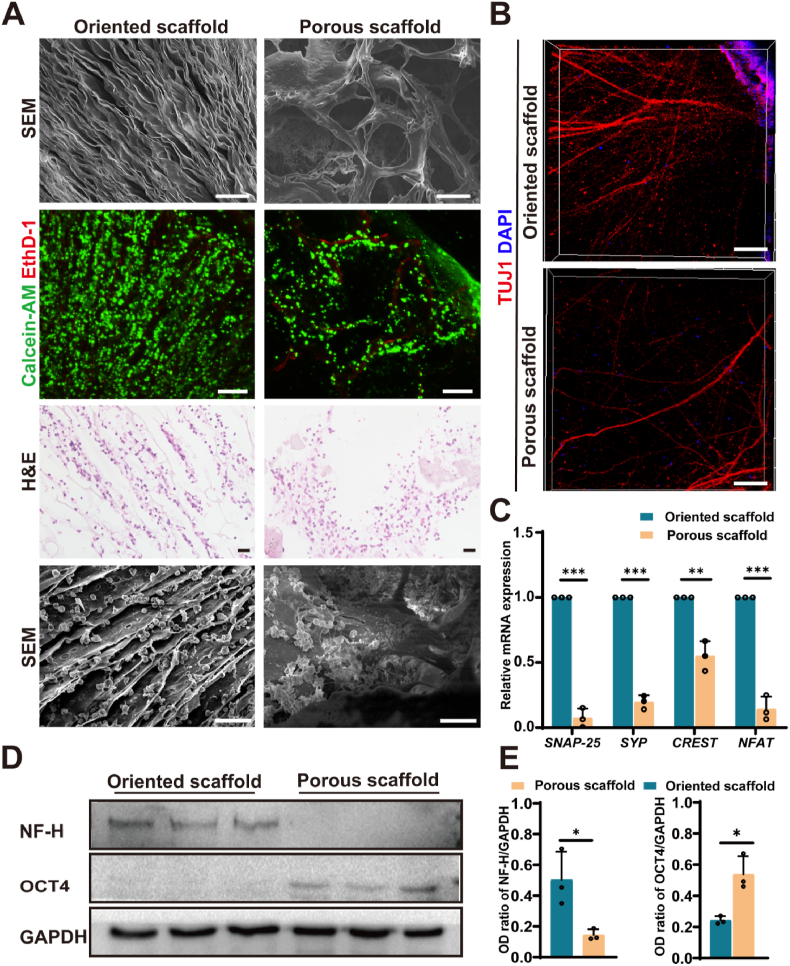
Structural, morphological, and functional comparison of oriented versus porous donut-shaped SF scaffolds in promoting differentiation of hiNSCs. (A) SEM images showing radially aligned pores in the oriented SF scaffold and randomly porous structures in the SF porous scaffold. Scale bar, 100 μm. Live/Dead staining shows high hiNSCs viability on both scaffold types. Cells aligned along radial pores on oriented scaffolds but appeared randomly distributed on conventional ones. Scale bar, 200 μm. H&E and SEM images showing radial alignment of hiNSCs on oriented scaffolds and aggregates on porous scaffolds. Scale bars: 20 μm (H&E), 50 μm (SEM). (B) Immunofluorescence images showing increased TUJ1-positive neurites in the central pore of oriented scaffolds, with uniform radial alignment toward the center compared to porous scaffolds. Scale bar, 100 μm. (C) RT-qPCR results showing higher expression of *SNAP25*, *SYP*, *CREST*, and *NFAT* in hiNSCs on oriented SF scaffolds (n = 3). (D–E) Representative Western blot images and quantification showing increased NF-H and decreased OCT4 expression in the oriented SF scaffold group. (n = 3). ∗*P* < 0.05,∗∗*P* < 0.01, ∗∗∗*P* < 0.001.

SEM revealed that the oriented SF scaffolds featured radially aligned pores formed by lamellar sheet-like structures, in contrast to the interconnected porous networks characteristic of conventional scaffolds. This unique architecture is anticipated to provide directional guidance for neurite extension. Accordingly, while both scaffold types supported comparable cellular viability for one week of culture *in vitro*, hiNSCs on the oriented scaffolds exhibited alignment along the radial pores, whereas those on conventional porous scaffolds displayed a random distribution. This distinctive cell alignment pattern was further corroborated by H&E staining, which showed that hiNSCs extended radially within the oriented scaffolds, while forming clustered aggregates within the pores of the conventional scaffolds. SEM images of hiNSCs-loaded scaffolds confirmed these observations. On oriented lamellar scaffolds, hiNSCs exhibited an elongated morphology aligned with the scaffold structure, with some cells migrating between lamellae. In contrast, porous scaffolds supported dense, clustered cell growth, consistent with H&E staining (Fig. 1A). These results highlight the influence of scaffold architecture on hiNSCs alignment and spatial distribution, with oriented structures guiding directional migration.

We next evaluated the phenotypic and functional characteristics of hiNSCs on different scaffolds. Compared to porous scaffolds, oriented scaffolds supported a greater number of β-tubulin(TUJ1)-positive neuronal projections within the central pore, with neurites exhibiting a more uniform, radial alignment extending from the scaffold toward the center (Fig. 1B). To assess neural differentiation at the molecular level, transcriptional expression of key neuronal markers, including calcium-responsive transactivator *(CREST)*, *NFAT*, synaptophysin *(SYP)*, and synaptosomal-associated protein 25 *(SNAP-25)* was quantified by RT-qPCR. All four genes were significantly upregulated in hiNSCs cultured on oriented scaffolds compared to those on porous scaffolds (Fig. 1C). Consistently, Western blot analysis revealed increased expression of neurofilament heavy chain (NF-H), a marker of neuronal maturation, along with decreased expression of the pluripotency marker octamer-binding transcription factor 4 (OCT4) in cells grown on oriented scaffolds (Fig. 1D and E). Together, these findings underscore the importance of scaffold architecture in guiding hiNSCs alignment, promoting directed neurite outgrowth, and enhancing neuronal differentiation.

### 4.2 hVOs incorporation promoted hiNSCs differentiation in SF scaffolds

hESC-derived hVOs were comprehensively characterized to confirm their structural and functional suitability for downstream co-culture and tri-culture applications (Fig. S2B–D). By day 12 of differentiation, immunostaining verified the presence of CD31-positive ECs, platelet-derived growth factor receptor β (PDGFRβ)-positive pericyte-like cells, and α-smooth muscle actin (αSMA)-positive smooth muscle-like cells. These cell populations self-organized into interconnected tubular networks with clearly defined lumens and basement membranes, as evidenced by H&E staining, COL4-positive immunofluorescence, and ultrastructural features observed via TEM. RT-qPCR further confirmed the vascular identity of hVOs, showing significant upregulation of endothelial (platelet endothelial cell adhesion molecule1 (*PECAM1*) and vascular endothelial cadherin (*VE-cadherin*)), mural (*PDGFRβ*), and smooth muscle (*αSMA*) markers relative to undifferentiated hESCs (Fig. S2E). These findings confirm the successful generation of vascularized hVOs that structurally and molecularly resemble native vascular tissue.

**Fig. 2:**
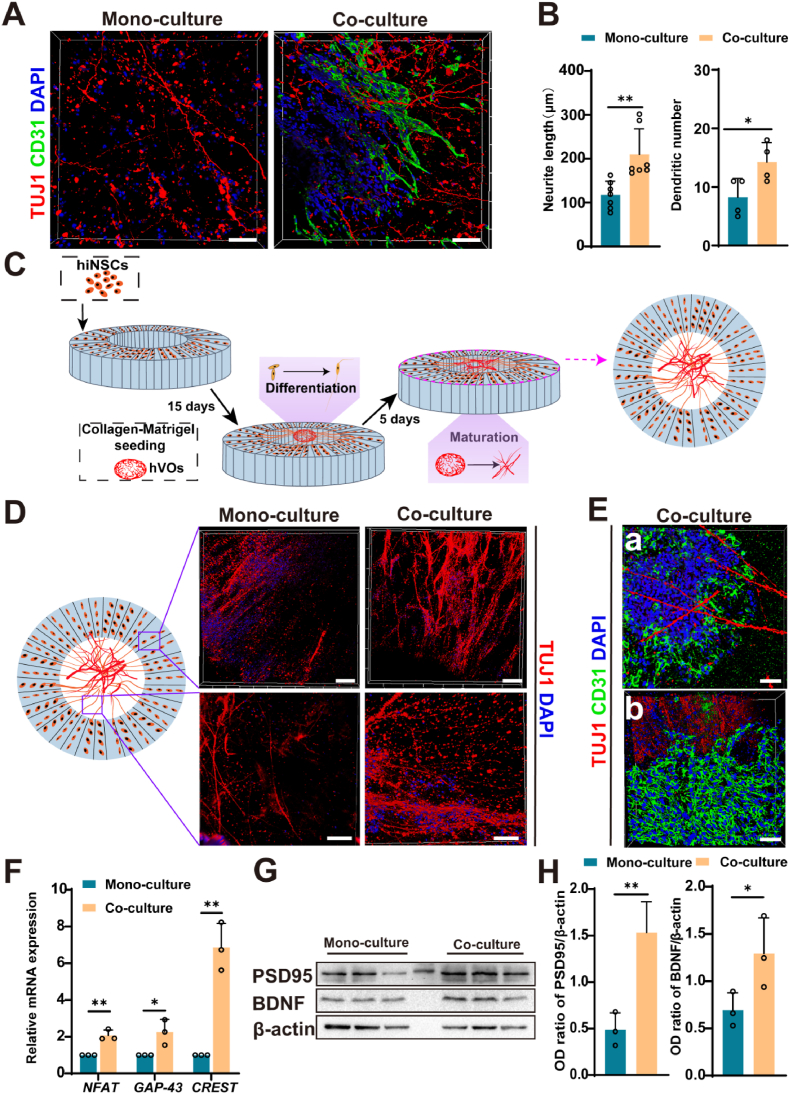
Enhanced neuronal differentiation and vascular-neuronal interactions in oriented donut-shaped SF scaffolds via hiNSCs and hVOs co-culture. (A) Immunofluorescence staining showing intermingled TUJ1-positive neurons (red) and CD31-positive hVOs (green) within the hydrogel-based co-culture. Scale bar, 100 μm. (B) Semi-quantitative analysis showing increased number and length of neuronal protrusions in the co-culture group compared to the mono-culture group (n = 7 and n = 4). (C) Pattern diagram showing the construction of a hiNSCs–hVOs co-culture model on a SF scaffold. (D) Representative immunofluorescence image showing increased neurite outgrowth in hiNSCs after co-culture with hVOs. Scale bar, 200 μm. (E) Spatial overlap of CD31-positive hVOs and TUJ1-positive neurons within the scaffold, with hVOs-derived structures extending outward from the central region along radial pores. Scale bar, 100 μm. (F) RT-qPCR analysis showing upregulated expression of *NFAT*, *GAP-43*, and *CREST* genes in the co-culture group compared to the mono-culture group (n = 3). (G–H) Representative Western blot images and quantification showing increased expression of BDNF and PSD95 proteins in the co-culture group (n = 3). *∗P<0.05, ∗∗P<0.01, ∗∗∗P<0.001*.

We next established a co-culture system within composite hydrogels to further investigate the influence of hVOs on hiNSCs differentiation. Immunofluorescence staining revealed that the capillary networks formed by hVOs were closely associated with the neuronal projections from differentiated hiNSCs, indicating direct physical interactions between the two cell types. Semi-quantitative analysis of the images showed that neurite outgrowth in the hiNSCs-hVOs co-culture group was significantly enhanced, with an average neurite length of 180 μm, approximately 1.3 times longer than in the hiNSCs mono-culture condition. Additionally, the number of dendrites was 1.7 times higher in the co-culture system compared to mono-culture (Fig. 2A and B). These data suggested the crucial role of hVOs in supporting hiNSCs differentiation and promoting neuronal maturation.

Building on these findings, a vascularized neuronal model using oriented donut-shaped SF scaffolds was then developed. The construction process is illustrated in Fig. 2C, where hiNSCs were seeded onto the scaffolds, while hVOs were introduced into the central pore. Immunofluorescence staining revealed that the number of TUJ1-positive neurons was significantly higher in the scaffold-based hiNSCs-hVOs co-culture system than in the hiNSCs mono-culture group after co-culturing with hVOs (Fig. 2D). Notably, the scaffold's radial pores guided the spatial organization of both CD31-positive tubular networks and TUJ1-positive neuronal projections, leading to an overlapping arrangement of vascular and neuronal structures. Moreover, the tubular structures of hVOs extended outward from the central region, integrating with the radial pores of the scaffold (Fig. 2E), confirming the feasibility of direct cell-cell interactions within the co-culture system.

To further evaluate the impact of hVOs on neuronal phenotype during SF scaffold-based culture, RT-qPCR analysis demonstrated a significant upregulation of neuronal growth and activity-related genes—such as growth-associated protein 43 (*GAP-43*), *NFAT*, and *CREST*—in the co-culture system compared to hiNSCs cultured alone (Fig. 2F). Moreover, scaffold-supported co-cultures exhibited significantly higher expression levels of neuronal markers, including *TUJ1* and *NFAT*, than hydrogel-based counterparts, highlighting the role of SF scaffolds in promoting neuronal maturation and validating their integration into the co-culture platform (Fig. S4). Consistently, Western blot analysis confirmed increased expression of brain-derived neurotrophic factor (BDNF) and postsynaptic density protein 95 (PSD95) in co-cultured hiNSCs relative to mono-cultures (Fig. 2G and H). Together, these findings indicate that hVOs, in conjunction with the SF scaffold, facilitate enhanced neuronal development and functional maturation within the donut-shaped co-culture system.

### 4.3 Distinct phenotypes of microglia differentially influence the differentiation of hiNSCs in SF scaffold

Prior to initiating the co-culture experiments, the successful polarization of HMC3 cells into distinct phenotypes was confirmed by Western blotting, immunofluorescence, and RT-qPCR analyses. M1 polarization was evidenced by elevated CD86 protein levels and upregulation of pro-inflammatory genes *IL-6* and *TNF-α*, whereas M2 polarization was validated by increased CD163 expression and enhanced transcription of *TGF-β1* and *CD206* (Fig. S5).

**Fig. 3:**
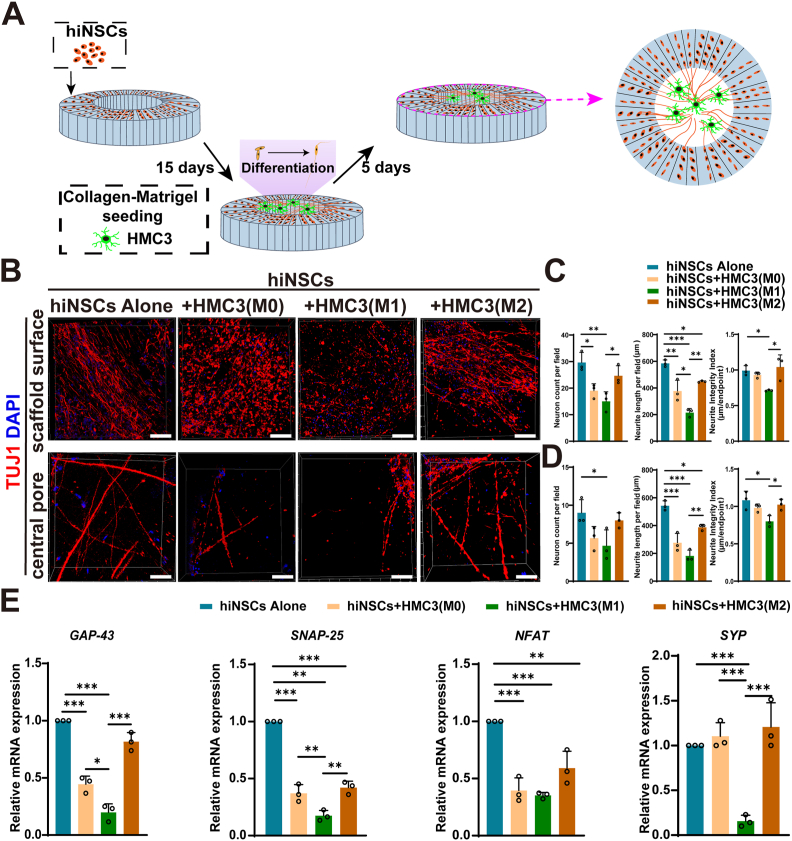
Differential impacts of distinct HMC3 microglial phenotypes on neuronal differentiation of hiNSCs within oriented SF scaffolds. (A) Schematic diagram for the construction of a neuron-microglia co-culture model on SF scaffold. (B) Immunofluorescence staining showing reduced neurite number and integrity in hiNSCs co-cultured with M1-polarized HMC3 cells, compared to M0, M2, and mono-culture groups. Scale bar, 100 μm. Quantification showing M1-polarized microglia impaired neuronal differentiation and network integrity in both scaffold surface (C) and central pore (D), while M2-polarized microglia preserved these features similar to mono-culture (n = 3). (E) RT-qPCR results showing reduced expression of neuronal markers (*SNAP-25*, *GAP-43, SYP*, and *NFAT*) in hiNSCs co-cultured with HMC3 cells, most notably in the M1 group (n = 3). *∗P<0.05, ∗∗P<0.01, ∗∗∗P<0.001*.

Following validation, the experimental workflow for co-culturing phenotypically distinct HMC3 cells with hiNSCs on the oriented SF scaffold was established, as illustrated in Fig. 3A. Compared to the hiNSCs mono-culture group, hiNSCs co-cultured with M1-polarized HMC3 cells exhibited a significant reduction in neurite number and structural integrity, whereas co-culture with M0 and M2 HMC3 had a less pronounced effect (Fig. 3B). This inhibitory influence of M1 polarization was consistently evident on both the scaffold surface and within the central pore region, where M1-polarized microglia most strongly impaired neuronal differentiation, neurite outgrowth, and network organization. By contrast, M0-polarized microglia caused only mild attenuation of these parameters, while M2-polarized microglia largely preserved neuronal differentiation and maintained a well-organized and structurally intact neuronal architecture, closely resembling mono-culture (Fig. 3C and D).

RT-qPCR analysis further demonstrated that co-culture with HMC3 cells led to a downregulation of neuronal differentiation markers, including *SNAP-25*, *GAP-43*, *SYP,* and *NFAT*, with the M1 phenotype exerting the strongest inhibitory effect (Fig. 3E). These results indicated that HMC3 suppresses hiNSCs differentiation in a phenotype-dependent manner, with M1-polarized HMC3 cells exhibiting the most pronounced inhibitory effect, whereas M2-polarized cells exerted the least suppression.

### 4.4 hVOs incorporation reverses M2 microglial suppression and promotes hiNSCs differentiation in SF scaffolds

**Fig. 4:**
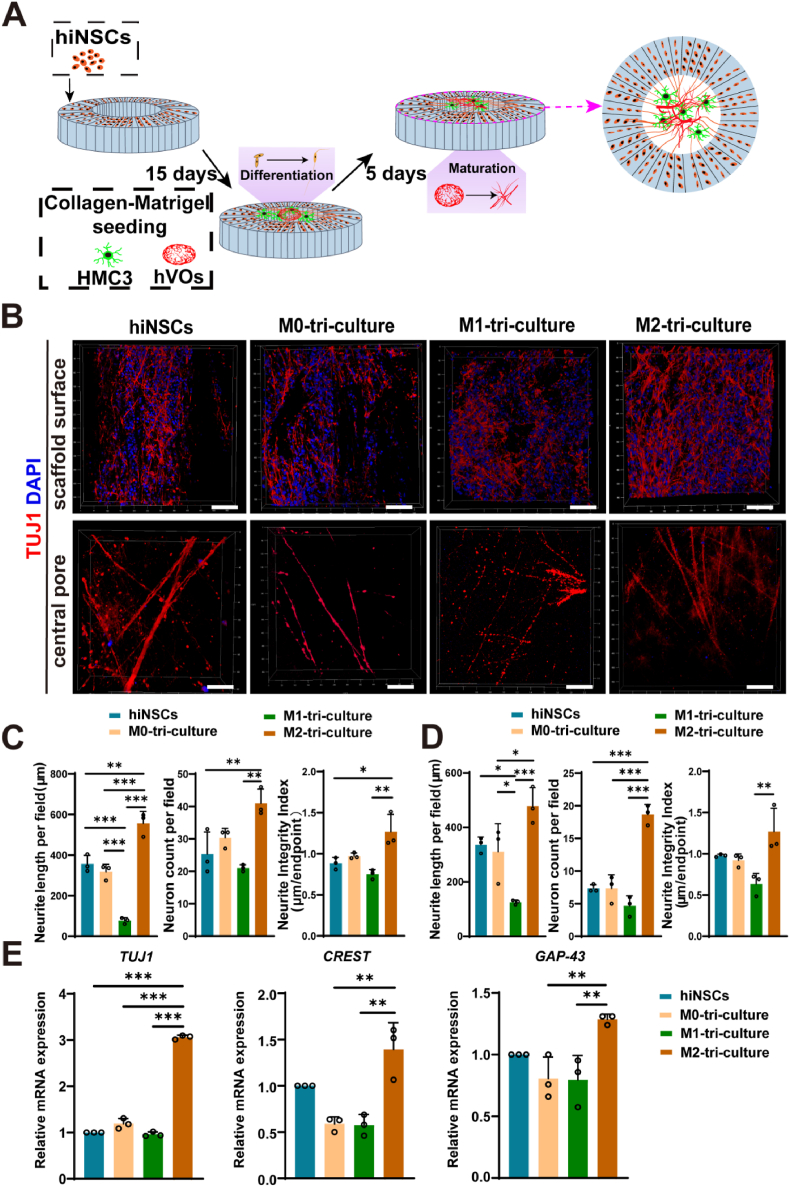
Characterization of the co-culture of different phenotypes of HMC3, hiNSCs, and hVOs in silk scaffolds. (A) Schematic illustration of the hiNSCs–hVOs–microglia tri-culture model on a SF scaffold. (B) Immunofluorescence staining showing dense and continuous TUJ1-positive neurons in the M2 tri-culture group, while the M1 group showed fewer, fragmented neurites. Scale bar, 100 μm. Semi-quantitative analysis on the scaffold surface (C) and within the central pore region (D), showing lowest neurite number, length, and continuity in the M1 tri-culture group; comparable levels in the M0 tri-culture and mono-culture; and highest values in the M2 tri-culture group. (n = 3). (E) RT-qPCR results showing upregulated expression of *TUJ1*, *CREST*, and *GAP-43* in the M2-tri-culture group compared to the hiNSCs mono-culture, M0, and M1 tri-culture groups (n = 3). *∗P<0.05, ∗∗P<0.01, ∗∗∗P<0.001*.

To further investigate whether the incorporation of hVOs influences neuron–microglia crosstalk, a tri-culture compartment comprising hiNSCs, HMC3 cells, and hVOs was established, as illustrated in Fig. 4A. Immunofluorescence staining revealed that, compared to the hiNSCs mono-culture, the M1-tri-culture exhibited a marked reduction in TUJ1-positive neurite number, shortened protrusion length, and increased fragmentation. In contrast, the M2 tri-culture markedly enhanced neurite outgrowth and structural continuity beyond levels observed in the mono-culture (Fig. 4B). Semi-quantitative analysis confirmed that these parameters were the lowest in the M1-tri-culture group. In contrast, the M0-tri-culture displayed neuronal morphology comparable to that of the monoculture. Notably, the M2-tri-culture significantly enhanced neurite number, extended neurite length, and improved continuity, achieving the highest scores among all groups (Fig. 4C and D). These morphological observations were corroborated by gene expression analyses, which demonstrated significant upregulation of neural differentiation markers (*TUJ1*, *CREST*, and *GAP-43*) in the M2-tri-culture group and suppression of these markers in the M1-tri-culture (Fig. 4E). Additionally, increased expression of PSD-95, a postsynaptic marker indicative of synaptic maturation, was observed in the M2-tri-culture (Fig. S6A), further supporting improved neuronal functional integration.

Collectively, these findings suggest that although M2-polarized HMC3 cells alone exert a mild inhibitory effect on hiNSCs differentiation (as described in Section 4.3), their co-culture with hVOs transforms this effect into a robust promotive signal, enhancing neurite outgrowth, elongation, and structural organization.

### 4.5 M2 HMC3 cells promote hVOs maturation via the SDF1/CXCR4 axis

**Fig. 5:**
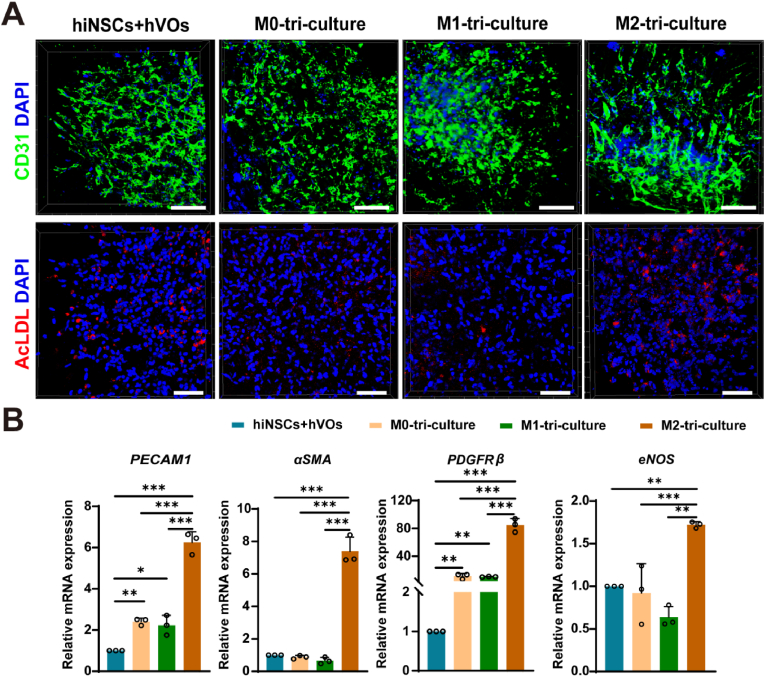
M2-polarized HMC3 cells promoting structural and functional maturation of endothelial components within hVOs. (A) Immunofluorescence staining of CD31 (green) showing M2-tri-culture exhibited greater network integrity, more branching, and longer vascular extensions compared with other conditions. Scale bar, 100 μm. DiI-Ac-LDL uptake assay (red) showing stronger and more extensive fluorescence in the M2 tri-culture group, indicating enhanced scavenger activity and endothelial functionality. Scale bar, 100 μm. (B) RT-qPCR analysis showing significantly increased expression of vascular-associated genes (*PECAM1*, *α-SMA*, *PDGFRβ*) and endothelial functional marker *eNOS* in the M2 tri-culture group compared with all other groups (n = 3). *∗P<0.05, ∗∗P<0.01, ∗∗∗P<0.001*.

In addition to the role of M2-polarized HMC3 cells in promoting hiNSCs differentiation, we further examined whether these cells also influence the maturation of hVOs within the tri-culture system. CD31-positive endothelial networks in the M2-tri-culture group displayed superior structural organization, characterized by increased branching, extended vessel length, and enhanced network integrity compared to other tri-cultures or the hiNSCs–hVOs co-cultures (Fig. 5A). Correspondingly, the expression of endothelial and pericyte-like mural cell markers—*PECAM1*, *αSMA*, and *PDGFRβ*—was significantly elevated, indicating enhanced vascular differentiation (Fig. 5B).

Functional assays further corroborated these findings. The M2-tri-culture group showed stronger and more extensive DiI-Ac-LDL uptake, suggesting increased scavenger activity, and endothelial nitric oxide synthase (*eNOS*) expression—a key marker of endothelial function—was markedly upregulated (Fig. 5A and B). Together, these results support the pivotal role of M2-polarized HMC3 cells in driving both structural and functional maturation of hVOs, emphasizing their contribution to a more physiologically relevant immune-vascular microenvironment.

**Fig. 6:**
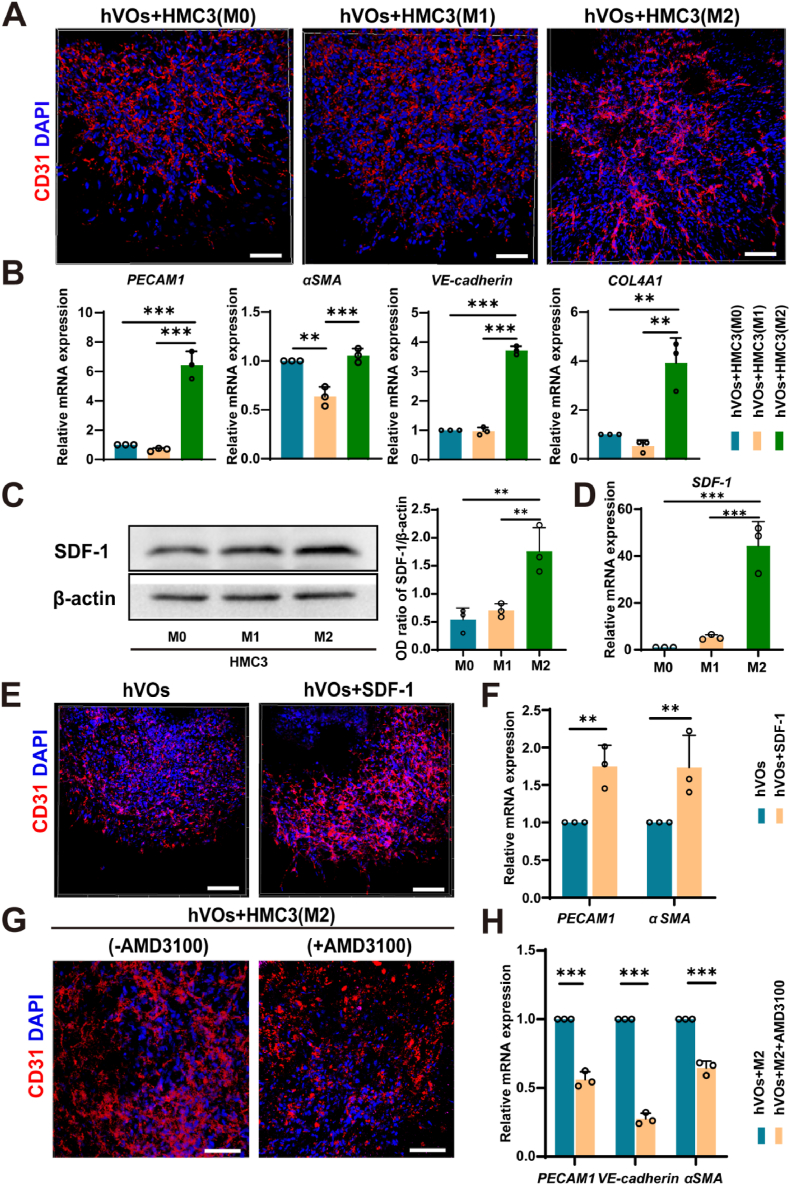
Effects of different HMC3 phenotypes and SDF-1/CXCR4 signaling on vascular network formation in hVOs. (A) CD31 immunofluorescence staining (red) showing increased number and length of vascular-like tubules in the hVOs–M2 HMC3 co-culture group compared to the hVOs–M1 HMC3 group. (B) RT-qPCR analysis showing increased expression of *PECAM1*, *VE-cadherin*, *αSMA*, and *COL4A1* in the hVOs-M2 HMC3 co-culture compared to the hVOs–M0 and hVOs–M1 HMC3 co-culture (n = 3). (C, D) Western blot and RT-qPCR analyses demonstrating *SDF-1* expression at both protein and mRNA levels was highest in M2-polarized HMC3 cells (n = 3). (E, F) Immunofluorescence and RT-qPCR analysis showing supplementation with recombinant SDF-1 enhanced vascular network formation and upregulated *PECAM1* and *αSMA* expression compared to the hVOs-alone group (n = 3). (G, H) Immunofluorescence and RT-qPCR results showing CXCR4 inhibition via AMD3100 disrupted CD31-positive vascular networks and downregulated *PECAM1*, *VE-cadherin*, and *αSMA expression* (n = 3). All scale bar, 100 μm ∗*P* < 0.05,∗∗*P* < 0.01, ∗∗∗*P* < 0.001.

To investigate the mechanism underlying M2 microglia-induced vascular maturation, we first analyzed single-cell RNA sequencing data from the human prefrontal cortex (GSE157827). The results revealed robust SDF-1–CXCR4 signaling between microglia and ECs under physiological conditions (Fig. S7–S8). Although the angiogenic role of this axis is well established [24,25], its specific involvement in microglia–endothelium crosstalk, particularly in the context of M2-polarized microglia acting on hVOs via SDF-1, has not been previously reported. These findings prompted us to examine further whether the SDF-1/CXCR4 axis functionally mediates the pro-angiogenic effects of M2-polarized microglia within our tri-culture model. Co-culture experiments revealed significantly enhanced vascular growth and elongation in the hVOs-M2 HMC3 co-culture group compared to the hVOs-M1 HMC3 co-culture group. Gene expression analysis further supported this observation, showing upregulation of vascular-related markers, including *PECAM1*, *VE-cadherin*, *α-SMA*, and *COL4A1*, in the hVOs-M2 HMC3 co-culture group. Consistently, M2-polarized HMC3 cells exhibited significantly elevated *SDF-1* expression at both the mRNA and protein levels compared to M0 and M1 phenotypes (Fig. 6A–D). Moreover, supplementation of recombinant SDF-1 significantly promoted vascular network formation and upregulated vascular marker expression, whereas pharmacological blockade of CXCR4 by AMD3100 markedly disrupted vascular integrity and reduced the expression of these markers in the hVOs-M2 HMC3 co-culture system (Fig. 6E–H). Together, these results identify M2-derived SDF-1 as a key mediator of vascular maturation in hVOs via the SDF1/CXCR4 signaling pathway.

**Fig. 7:**
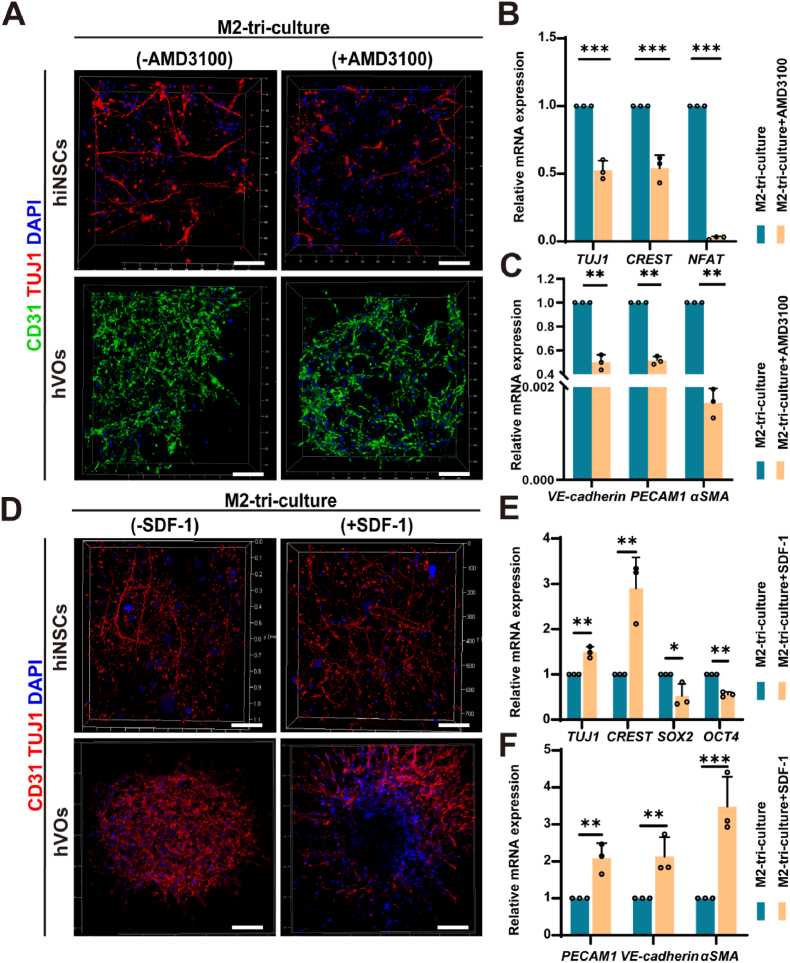
SDF-1/CXCR4 signaling enhances neurovascular differentiation in the M2-type HMC3 tri-culture model. (A) Immunofluorescence staining showing that CXCR4 inhibition by AMD3100 disrupted the formation of TUJ1-positive neuronal networks and CD31-positive capillary structures within hVOs in the M2 tri-culture system. (B,C) RT-qPCR results showing reduced expression of neuronal differentiation markers (*TUJ1*, *CREST*, *NFAT*) and vascular-associated genes (*VE-cadherin*, *PECAM1*, *α-SMA*) in the AMD3100-treated M2 tri-culture group compared to untreated controls (n = 3). (D) Immunofluorescence staining showing SDF-1 supplementation enhanced TUJ1-positive neuronal networks and CD31-positive vascular tubules in the M2 tri-culture. (E, F) RT-qPCR analysis showing that SDF-1 supplementation in the M2 tri-culture model upregulated neuronal (*TUJ1*, *CREST*) and vascular (*PECAM1*, *αSMA, VE-cadherin*) genes, while downregulating stemness markers (*SOX2*, *OCT4*) (n = 3). All scale bar, 100 μm ∗*P* < 0.05, ∗∗*P* < 0.01, ∗∗∗*P* < 0.001.

Given our findings suggesting that the SDF-1/CXCR4 signaling axis might play a key role in mediating the pro-developmental effects of M2-polarized HMC3 cells on hVOs, we next investigated how hVOs incorporation not only mitigated the inhibitory effects of M2-HMC3 cells but also enhanced the differentiation of hiNSCs. To explore this interaction, the M2-tri-culture system was treated with 5 μM AMD3100. This intervention disrupted the formation of TUJ1-positive neuronal networks and CD31-positive capillary structures within hVOs, as shown in Fig. 7A. Additionally, the expression of neuronal differentiation markers (*TUJ1, CREST*, *NFAT*) and vascular-related genes (*PECAM1*, *VE-cadherin*, *αSMA*) in hVOs was significantly downregulated (Fig. 7B and C). In contrast, Supplementation with recombinant SDF-1 (100 ng/mL) restored the density and integrity of both TUJ1-positive neuronal networks and CD31-positive capillary structures. SDF-1 treatment also led to upregulation of neuronal differentiation markers *TUJ1* and *CREST*, while concurrently downregulating pluripotency markers *SOX2* and *OCT4*. Moreover, vascular maturation-related genes (*PECAM1*, *VE-cadherin*, and *αSMA*) were also upregulated following SDF-1 supplementation (Fig. 7D–F).

**Fig. 8:**
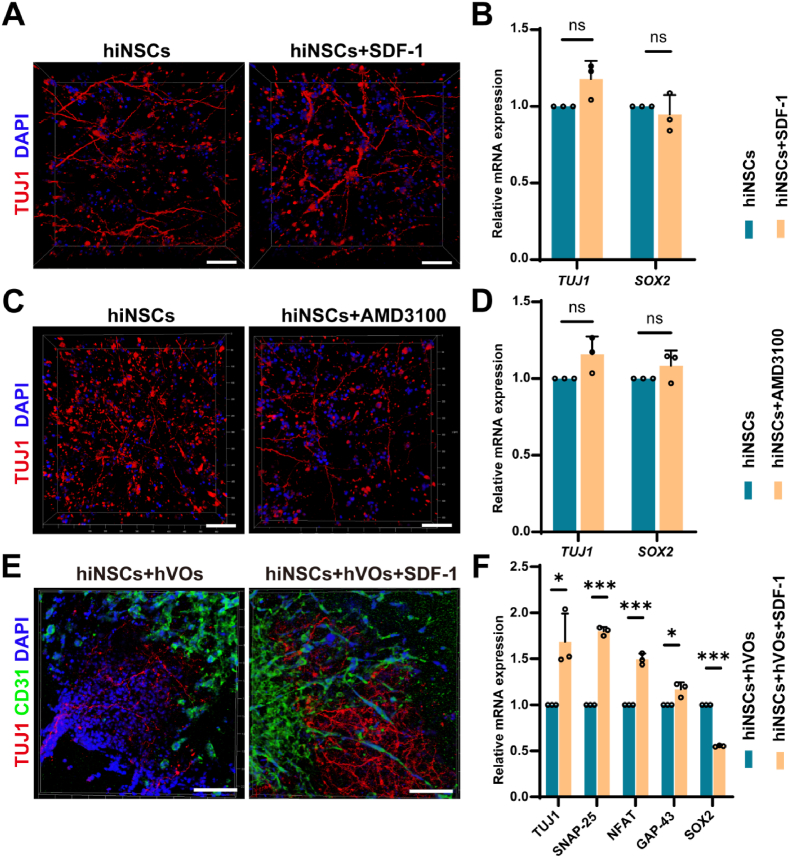
Differential effects of SDF-1/CXCR4 signaling on hiNSCs mono-culture and co-culture neurovascular organization (A-D) Immunofluorescence staining of TUJ1 and RT-qPCR analysis of *TUJ1* and *SOX2* expression showing no significant differences among untreated, SDF-1-treated, and AMD3100-treated hiNSCs groups (n = 3). Scale bar, 100 μm. (E) Immunofluorescence staining showing SDF-1 supplementation in the hiNSCs–hVOs co-culture enhanced TUJ1-positive neuronal network density, alignment, and neurite extension, alongside improved organization of CD31-positive vascular-like structures.Scale bar, 500 μm. (F) RT-qPCR analysis of neuronal maturation and synaptic markers (*TUJ1*, *SNAP-25*, *NFAT*, *GAP-43*) showing significant upregulation upon SDF-1 treatment, while the stemness gene *SOX2* downregulated (n = 3). ∗*P* < 0.05, ∗∗∗*P* < 0.001.

Notably, treatment with either SDF-1 or AMD3100 produced no observable effect on neuronal morphology or the gene expression of *TUJ1* and *SOX2* in hiNSCs cultured alone (Fig. 8A–D). In contrast, when SDF-1 was added to the hiNSCs-hVOs co-culture system, TUJ1-positive neuronal networks became more abundant and exhibited enhanced alignment and neurite extension. Simultaneously, CD31-positive vascular-like structures showed improved organization. These morphological changes were accompanied by a significant upregulation of neuronal maturation and synaptic markers (*TUJ1, SNAP-25, NFAT,* and *GAP-43*), along with a marked downregulation of the stemness marker *SOX2*, indicating enhanced neuronal differentiation in response to SDF-1 (Fig. 8E and F). These findings suggest that the SDF-1/CXCR4 signaling axis plays a pivotal role in promoting the maturation and differentiation of both hVOs and hiNSCs. However, this effect is not exerted directly on hiNSCs. Instead, M2-polarized HMC3 cells activate the SDF-1/CXCR4 pathway to promote hVOs maturation, which subsequently supports and enhances hiNSCs differentiation within the M2-based tri-culture system.

### 4.6 M2 HMC3 cells enhance BDNF expression in hVOs and accelerate hiNSCs differentiation

To identify potential molecular mediators contributing to the synergistic effect of M2-polarized HMC3 cells and hVOs on hiNSCs differentiation, we first performed a screening analysis using the publicly available single-cell RNA-sequencing dataset (GSE157827). Venn diagram analysis identified five genes (*BDNF*, *SNAP25*, *SLC6A1*, *SLC1A2*, and *NSF*) that were co-expressed in both ECs and neural stem cells (NSCs) within the normal human brain (Fig. S8E). Among these candidates, BDNF was of particular interest due to its well-established role as a neurotrophic factor regulating neuronal survival, growth, and differentiation.

**Fig. 9:**
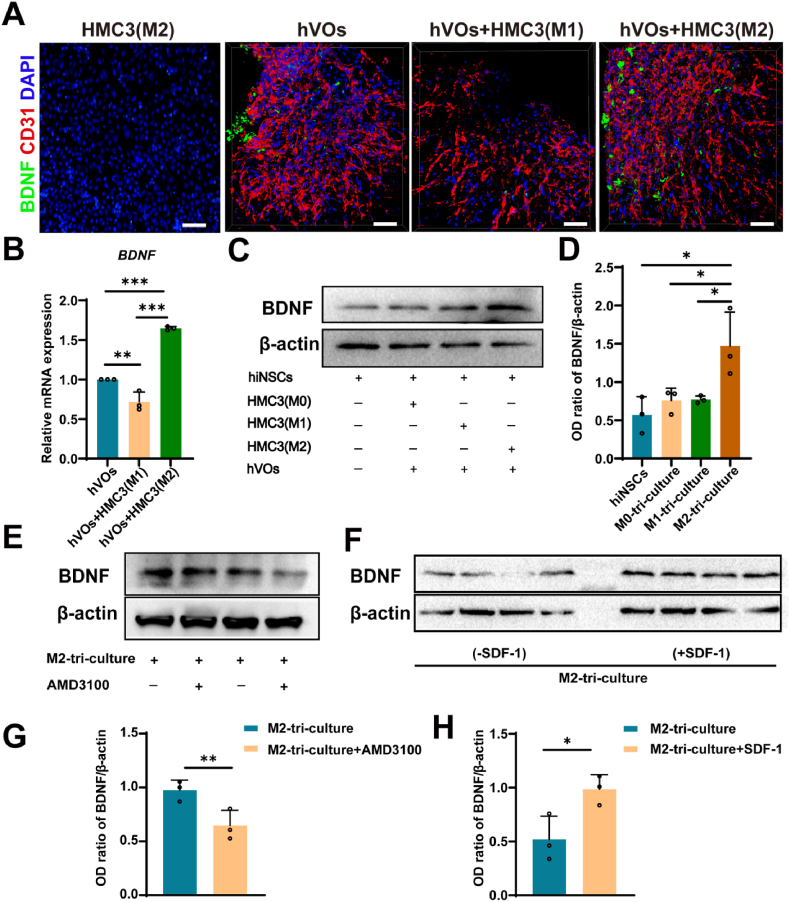
M2-polarized HMC3 cells induce BDNF expression in mature hVOs within a vascularized tri-culture model (A-B) Immunofluorescence staining and RT-qPCR analysis showing significantly elevated BDNF expression in the hVOs–M2 HMC3 co-culture group compared to the hVOs–M1 HMC3 group (n = 3). Scale bar, 100 μm. (C–D) Western blot analysis demonstrating elevated BDNF protein levels in the M2-tri-culture group compared to M1-and M0-tri-culture groups (n = 3). (E, G) Western blot images and semi-quantitative analysis showing AMD3100-mediated CXCR4 inhibition reduced BDNF expression in the M2 tri-culture system (n = 3). (F, H) Western blot images and semi-quantitative analysis showing increased BDNF expression following SDF-1 supplementation in the M2-tri-culture group compared with untreated control (n = 3). ∗*P* < 0.05, ∗∗*P* < 0.01, ∗∗*P < 0.001.*

Accordingly, immunofluorescence staining confirmed that BDNF-positive cells were significantly increased in the hVOs-M2 HMC3 co-culture compared to the hVOs-M1 HMC3 co-culture, suggesting that M2-polarized microglia enhance neurotrophic support. Further analysis revealed that *BDNF* gene expression was significantly upregulated in the hVOs–M2 HMC3 co-culture group, implicating BDNF as a key mediator of the pro-differentiation effects observed. Consistently, Western blot analysis showed markedly elevated BDNF protein levels in the M2 HMC3 tri-culture compared to the M1 group (Fig. 9A–D). Notably, treatment with the CXCR4 antagonist AMD3100 led to a reduction in BDNF expression, while supplementation with recombinant SDF-1 substantially increased BDNF levels (Fig. 9E–H). These results suggest that M2-polarized HMC3 cells promote hVOs maturation via the SDF-1/CXCR4 signaling axis, and the functionally enhanced hVOs, in turn, secrete BDNF, which facilitates the maturation and neuronal differentiation of hiNSCs.

## 5 Discussion

In this study, we developed an improved vascularized neuron-microglia tri-culture system by integrating hiNSCs, hVOs, and microglial cells within an optimized donut-shaped SF scaffold with a collagen-Matrigel hydrogel supplementation. To enable spatially defined cellular organization and interactions, hiNSCs were seeded along the scaffold's outer ring, while hVOs and microglia were localized within its central pore. This geometrically guided arrangement effectively mimics the anatomical distribution of cortical gray and white matter, with neural components positioned in the outer (gray matter-like) region and vascular/immune elements concentrated in the central (white matter-like) core. This configuration promotes directed axonal outgrowth toward the center and enables investigation of neurovascular and neuroimmune interactions within a structurally defined neural tissue model. Compared to conventional *in vitro* vascularized neuronal culture models, this 3D tri-culture platform offers enhanced structural fidelity, a more physiologically relevant microenvironment, and improved cell–cell interactions, providing a robust tool for studying the dynamic crosstalk of the neurovascular microenvironment. Several key advantages, including enhanced structural complexity, a more physiologically relevant microenvironment, and improved cellular interactions that better recapitulate *in vivo* neurovascular dynamics.

In particular, by employing a donut-shaped silk scaffold with radially aligned pores, we effectively directed axonal extension and promoted the formation of white matter-like structures within the hydrogel core, thereby significantly enhancing the neuronal differentiation of hiNSCs. Compared to a previously reported porous silk scaffold-based neural model [19], this design supported the development of longer and more oriented axonal projections and facilitated the establishment of synaptic networks. These findings, consistent with prior studies [[26], [27], [28], [29]], emphasize the pivotal role of scaffold topography in orchestrating axonal organization and neuronal maturation. In addition, to overcome the vascularization challenge in neural models, hESCs-derived hVOs were integrated into the tri-culture system. Unlike traditional endothelial cell-only models [30,31], hVOs self-organized into capillary-like networks containing ECs, pericytes, and smooth muscle cells, forming a more physiologically relevant vascular niche. This niche not only guided axonal alignment but also secreted neurotrophic and neurogenic cues that significantly promoted hiNSCs differentiation. These findings underscore the dual function of vascular networks as structural scaffolds and active regulators of neurogenesis.

To support the physiological relevance of our experimental model and guide mechanistic exploration, a transcriptomic data mining using publicly available single-cell RNA-sequencing data from the human prefrontal cortex. And our analysis highlighted prominent ligand-receptor interactions between microglia and endothelial cells, with the SDF-1/CXCR4 signaling axis emerging as a potential mediator of vascular communication. In parallel, a set of neurogenic factors, including BDNF, that were commonly expressed in both endothelial cells and neural stem cells, was identified, suggesting that the vascular compartment may contribute to neurotrophic support. These insights provided a rationale for targeting SDF-1/CXCR4 signaling and BDNF production in our tri-culture model to investigate their functional roles in coordinating vascular maturation and hiNSCs differentiation.

Mechanistically, the SDF-1/CXCR4 axis emerged as a key regulatory pathway linking vascular remodeling to neurogenesis. While traditionally recognized for its roles in angiogenesis and NSC migration [32,33], SDF-1/CXCR4 signaling in our system directly mediates hiNSCs differentiation through M2-polarized microglia-derived SDF-1 activation of CXCR4 in hVOs, driving vascular network maturation and the upregulation of angiogenesis-related markers. Inhibition of CXCR4 abrogated these effects, confirming the mechanistic coupling between angiogenesis and neurogenesis. Previous studies have shown that the SDF-1/CXCR4 axis plays multifaceted roles in promoting angiogenesis, enhancing vascular stability, regulating blood–brain barrier maturation, and supporting vascular remodeling and repair [34,35]. Additionally, it has been implicated in modulating neural stem cell function within the central nervous system, further suggesting a neuroprotective role of endogenous M2-type microglia. This underscores the physiological significance of SDF-1/CXCR4 as a bidirectional mediator of neurovascular integration.

Among downstream effectors, BDNF is identified as a pivotal molecule linking vascular function to neuronal fate. As a canonical neurotrophin, BDNF plays essential roles in neuronal survival, differentiation, and synaptic plasticity [36,37]. Functional analysis of the tri-culture model revealed that SDF-1/CXCR4 activation significantly upregulated BDNF expression in hVOs, whereas CXCR4 inhibition markedlyreduced it. These findings support a model in which SDF-1-driven vascular remodeling enhances the neurotrophic function of hVOs, thereby facilitating hiNSCs differentiation. This is consistent with previous reports demonstrating that M2-polarized microglia promote neurogenesis and vascular remodeling through secreting trophic factors such as SDF-1 and BDNF [[38], [39], [40]]. Notably, recent studies also suggest that M2-derived exosomes target ECs to enhance NSCs differentiation [41], further supporting a microglia-endothelium-neuron regulatory axis.

Our findings align with and extend previous studies showing that M2-polarized microglia support angiogenesis and neurogenesis through the secretion of trophic factors [38,42]. Beyond these classical roles, we demonstrate that M2 microglia further promote vascular remodeling and facilitate NSCs differentiation by maintaining neurovascular niche homeostasis. In particular, M2 microglia have been shown to regulate vascular maturation and neurogenesis via both exosome-mediated signaling and SDF-1 secretion [39,40] Consistently, recent studies revealed that M2 microglia-derived exosomes directly target endothelial cells, thereby enhancing NSCs differentiation and neurogenesis [41]. Together, these results underscore the pivotal role of M2-polarized microglia as key orchestrators of neurovascular remodeling and NSCs fate determination, and may offer promising targets for neurovascular regeneration. Besides, mechanisms of interaction between the phenotypic heterogeneity of microglia and the neurovascular microenvironment have demonstrated significant scientific value in *in vitro* research models. Such phenotypic diversity contributes to varying experimental outcomes, which have been attributed to differences in cell line origins, co-culture configurations, and the distinct functional polarization states of microglia [[43], [44], [45]]. These observations further highlight the importance of systematically evaluating microglial phenotype-dependent effects in neurovascular models.

Despite these important findings, the precise cellular source of BDNF within hVOs remains to be conclusively identified. Although endothelial cells are hypothesized to be the primary contributors, based on previous studies implicating endothelial-derived BDNF in neurovascular coupling and neuroprotection [46,47], contributions from other vascular components—such as pericytes or smooth muscle cells—cannot be excluded. Moreover, the roles of other endothelial-derived neurotrophic factors, including NGF, HGF, and VEGF, and their potential crosstalk with BDNF signaling, remain largely unexplored [48,49].

It should also be noted that, while the tri-culture model developed in this study offers notable advantages for simulating immune–neurovascular interactions *in vitro*, it still has limitations in fully recapitulating the complexity of the *in vivo* brain microenvironment. On one hand, the spatially compartmentalized seeding strategy enhances model observability and facilitates cell type-specific analyses; however, it limits direct physical contact between vascular or immune cells and neuronal cell bodies localized on the scaffold ring, thereby compromising the model's biomimetic fidelity. On the other hand, the absence of astrocytes—key components of the BBB—may limit the model's capacity to fully capture certain aspects of in *vivo* neurovascular physiology. To address these limitations, future studies will incorporate human astrocytes to develop a more biomimetic *in vitro* model that better recapitulates the BBB within the immune-neurovascular microenvironment. Although increased cellular complexity may pose challenges for downstream cell type-specific analyses, the application of spatial transcriptomics and advanced imaging techniques will enable comprehensive, dissociation-free mapping and characterization of heterogeneous cell populations. These improvements are expected to yield a more physiologically relevant neural tissue model, capable of elucidating the complex interplay among immune, neural, and vascular components.

**In summary,** although some limitations remain, this study establishes a spatially defined tri-culture model that enables controlled interrogation of immune-neuron-vascular interactions *in vitro*. These findings provide a foundation for future investigations into the cellular crosstalk underlying brain development, disease progression, and therapeutic modulation of neuroimmune and neurovascular dynamics.

## 6 Conclusion

Reconstructing the intricate architecture of the brain microenvironment remains a key challenge in neural tissue engineering. In this study, we developed a spatially organized tri-culture system by integrating hiNSCs, hVOs, and HMC3 microglia within a bioengineered silk scaffold, thereby recapitulating essential structural and cellular components of the immune-neurovascular microenvironment *in vitro*. This advanced model enabled controlled investigation of microglia–vascular–neural interactions and provided a physiologically relevant model for dissecting neurovascular mechanisms. Notably, we identified the SDF-1/CXCR4 signaling axis as a critical mediator of vascular-driven neuronal differentiation and demonstrated phenotype-specific effects of microglia on both hiNSCs maturation and hVOs development. Together, these findings present a robust platform for investigating neurovascular integration and establish a foundation for future translational applications in neuroregenerative medicine.

## CRediT authorship contribution statement

**Yinhe Han:** Writing – original draft, Formal analysis, Data curation. **Lina Guo:** Formal analysis, Data curation. **Mingqi Wang:** Data curation. **Zhen Cao:** Data curation. **Xu Zheng:** Data curation. **Xinyu Wang:** Data curation. **Lingling Jin:** Data curation. **Xiaoqing Wei:** Data curation. **Xiuli Wang:** Writing – review & editing, Supervision, Funding acquisition, Conceptualization. **Jie Zhao:** Writing – review & editing, Supervision, Funding acquisition, Conceptualization.

## Ethics approval and consent to participate

This study did not involve human participants or animal experiments; hence, ethics approval and consent to participate were not required.

## Declaration of competing interest

The authors declare no conflict of interest.
