# Supplementary material for: Bioengineering an improved three-dimensional vascularized co-culture model for studying Neuron–Microglia interactions
Source: Bioact Mater. 2025 Sep 10;54:813–28. doi: 10.1016/j.bioactmat.2025.09.008 (PMC12628055; doi:10.1016/j.bioactmat.2025.09.008)
Supplement: Multimedia component 1 [file mmc1.docx]

**Supplementary method:**

**Single-cell RNA sequencing data processing and analysis**

**Data acquisition and preprocessing**

Single-cell transcriptomic data of the prefrontal cortex from 9 cognitively normal individuals and 12 Alzheimer's disease (AD) patients were obtained from the Gene Expression Omnibus (GEO) database under accession number GSE157827.^1^ The raw UMI count matrices were imported into the R environment (version 4.3.2) using the Seurat package (version 4.2.0). To account for batch effects across the 21 samples, we applied the Harmony integration algorithm. Cells expressing fewer than 200 genes or with >20% mitochondrial gene-derived transcripts were excluded from downstream analyses. After filtering, data normalization was performed using a global-scaling log-normalization method (NormalizeData function). Highly variable genes (HVGs, n=2000) were identified using the FindVariableFeatures function.

**Dimensionality reduction, clustering, and cell-type annotation**

Principal component analysis (PCA) was performed on the integrated dataset using the RunPCA function, retaining the top 30 principal components for downstream analysis. A shared nearest neighbor (SNN) graph was constructed via the FindNeighbors function, followed by unsupervised clustering using the FindClusters function with a resolution set to 2.5. Differentially expressed genes (DEGs) across clusters were identified with the FindAllMarkers function under default parameters. DEGs were defined as genes with an adjusted p-value < 0.05 (Benjamini–Hochberg correction) and a minimum fold change≥2(|log2FC|≥1). Cluster identities were manually assigned by referencing established marker gene expression profiles reported in prior studies. The major cell types and corresponding marker genes were as follows:

Neural stem cells (NSCs)-*CXCR4*, *VIM*, *MSI1*, *SOX2*, *NES*. Astrocytes-*SLC1A2*, *ADGRV1*, *GPC5*, *RYR3*, *GFAP*. Endothelial cells (ECs)-*CLDN5*, *FLT1*, *ABCB1*, *EBF1*, *MT2A*. Excitatory neurons-*RALYL*, *KCNIP4*, *CBLN2*, *LDB2*, *KCNQ5*. Inhibitory neurons-*NXPH1*, *LHFPL3*, *PCDH15*, *GRIK1*, *ADARB2*. Microglia-*LRMDA*, *DOCK8*, *ARHGAP24*, *ARHGAP15*, *PLXDC2*. Oligodendrocytes-*ST18*, *PLP1*, *CTNNA3*, *MBP*, *PIP4K2A.*

**Cell-cell communication analysis**

To infer potential intercellular communication networks, we employed the CellChat package (version 1.6.1). The analysis was performed separately for the control and AD groups. For each group, cell-cell communication probability was estimated based on the expression of known ligand-receptor pairs curated from the CellChatDB human interaction database. To assess the statistical significance of each ligand-receptor interaction, a permutation test was performed by randomly permuting cluster labels 1000 times to generate a null distribution. The empirical p-value for each interaction was calculated as the proportion of permutations where the average interaction strength exceeded the observed value. Significant interactions were visualized and compared between control and AD groups to explore disease-associated alterations in intercellular communication.

1. Lau, S. F.; Cao, H.; Fu, A. K. Y.; Ip, N. Y., Single-nucleus transcriptome analysis reveals dysregulation of angiogenic endothelial cells and neuroprotective glia in Alzheimer's disease. *Proc Natl Acad Sci U S A.* **2020,** *117* (41), 25800-25809.
