# Supplementary material for: Bioengineering an improved three-dimensional vascularized co-culture model for studying Neuron–Microglia interactions
Source: Bioact Mater. 2025 Sep 10;54:813–28. doi: 10.1016/j.bioactmat.2025.09.008 (PMC12628055; doi:10.1016/j.bioactmat.2025.09.008)
Supplement: Multimedia component 2 [file mmc2.docx]

**Supplementary figures(Fig. S1-S8):**

**Fig. S1**


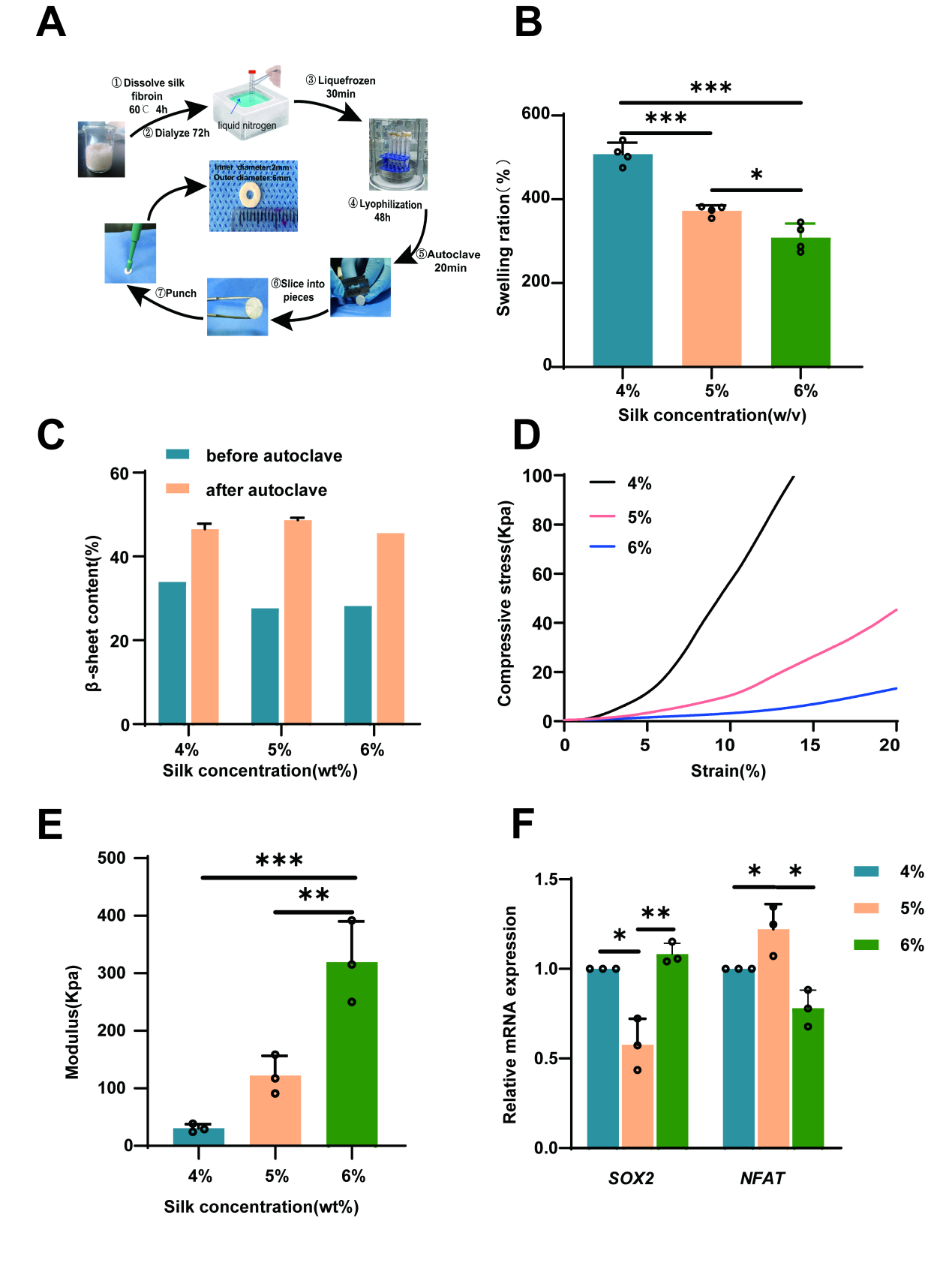


Physical and biological characterization of SF scaffolds at concentrations of 4%, 5%, and 6% (w/v).

(A) Schematic illustration of the fabrication process for oriented SF. (B) Swelling ratios of scaffolds fabricated at different SF concentrations (4%, 5%, 6%) (n=4). (C) β-sheet content of scaffolds before and after autoclaving, assessed by FTIR analysis. (D) Stress–strain curves from unconfined compression testing of hydrated scaffolds. (E) Quantification of compressive modulus across SF concentrations. (F) RT-qPCR results showing the lowest *SOX2* and highest *NFAT* expression levels in 5% SF scaffolds (n = 3). **P* < 0.05, ***P* < 0.01, ****P* < 0.001.

**Fig. S2**


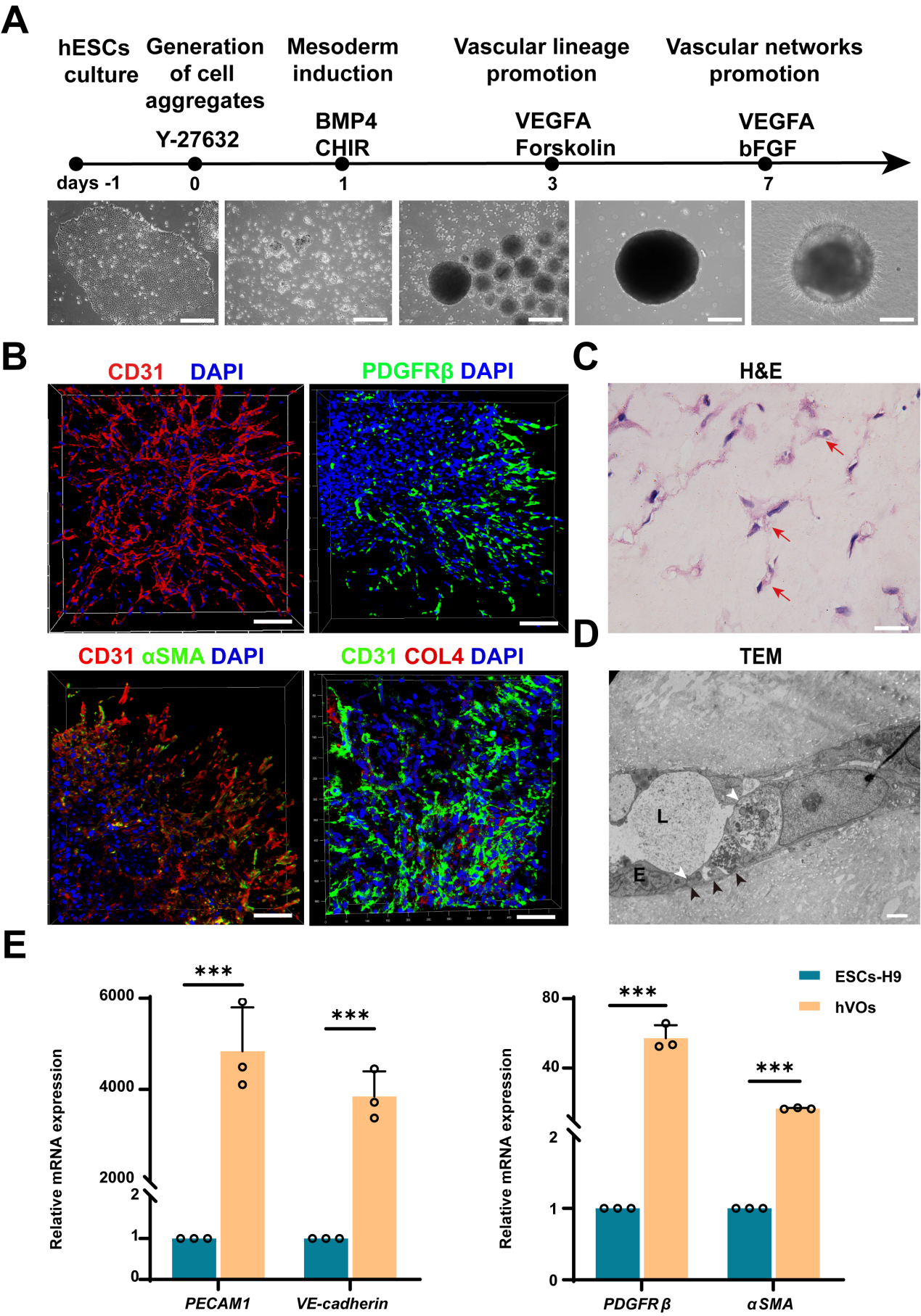


Generation of hVOs from hESCs.

(A) Stepwise differentiation protocol for generating hVOs from hESCs. Scale bar, 100 μm. (B) Representative immunofluorescence images showing vascular marker expression in induced hVOs. CD31-positive endothelial cells (red) form dense vascular-like networks. PDGFRβ-positive cells (green) suggesting the presence of mural cells such as pericytes. Co-staining of αSMA (green) and CD31 (red), showing smooth muscle-like cells adjacent to endothelium. Collagen IV (COL4, red) deposition around CD31-positive vessels (green), indicating basement membrane formation. Scale bar, 100 μm. (C) H&E staining showing lumen-like structures (red arrows) formed within hVOs. Scale bar, 20 μm. (D) TEM image showing lumen-like ultrastructural morphology within hVOs. Note the generation of lumenized, the appearance of tight junctions (white arrowheads) and a basement membrane (black arrowheads). L, lumen; E, endothelial cell. Scale bar, 2 μm. (E) RT-qPCR analysis of vascular-related genes, including *PECAM1*, *VE-cadherin*, *PDGFRβ*, and *αSMA*, showing significantly higher expression in hVOs compared to undifferentiated hESCs (n=3). ****P* < 0.001.

**Fig. S3**


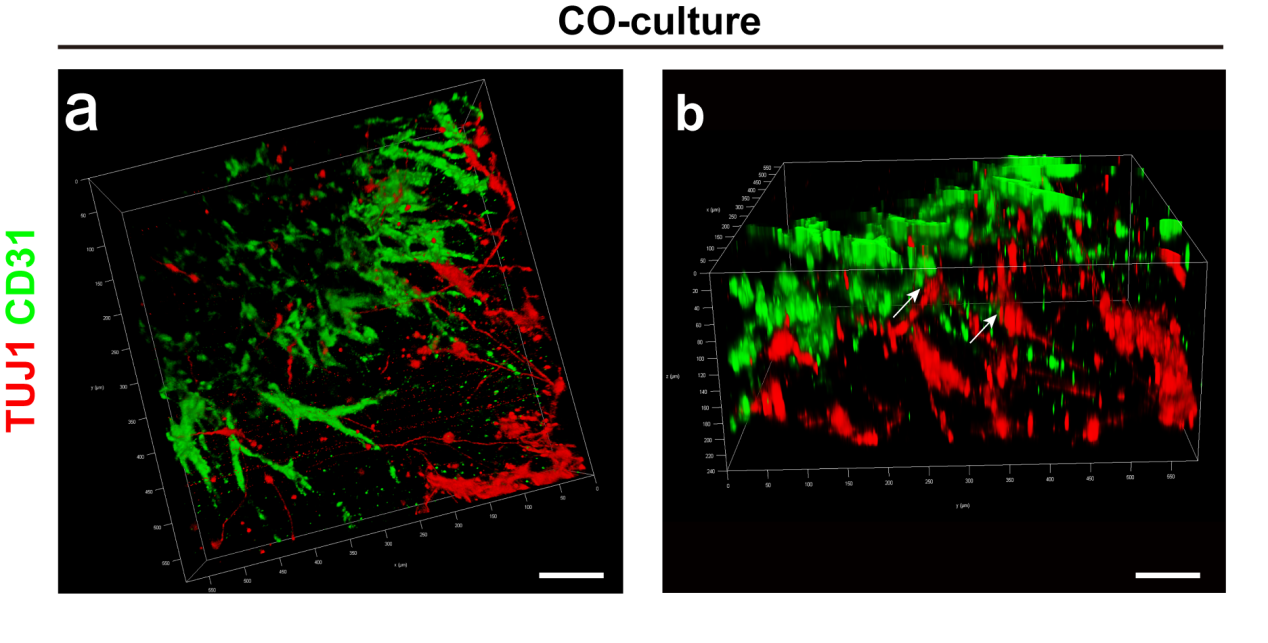


Neurovascular spatial relationships in hVOs.

Three-dimensional confocal reconstructions illustrating the spatial interactions between TUJ1-positive neurons (red) and CD31-positive endothelial cells (green). (a) Isometric view revealing the overall neurovascular architecture. (b) Orthogonal projection highlighting close juxtaposition between neuronal processes and CD31-positive vessel-like structures, indicating potential neurovascular interfaces. Scale bar, 100 μm.

**Fig. S4**


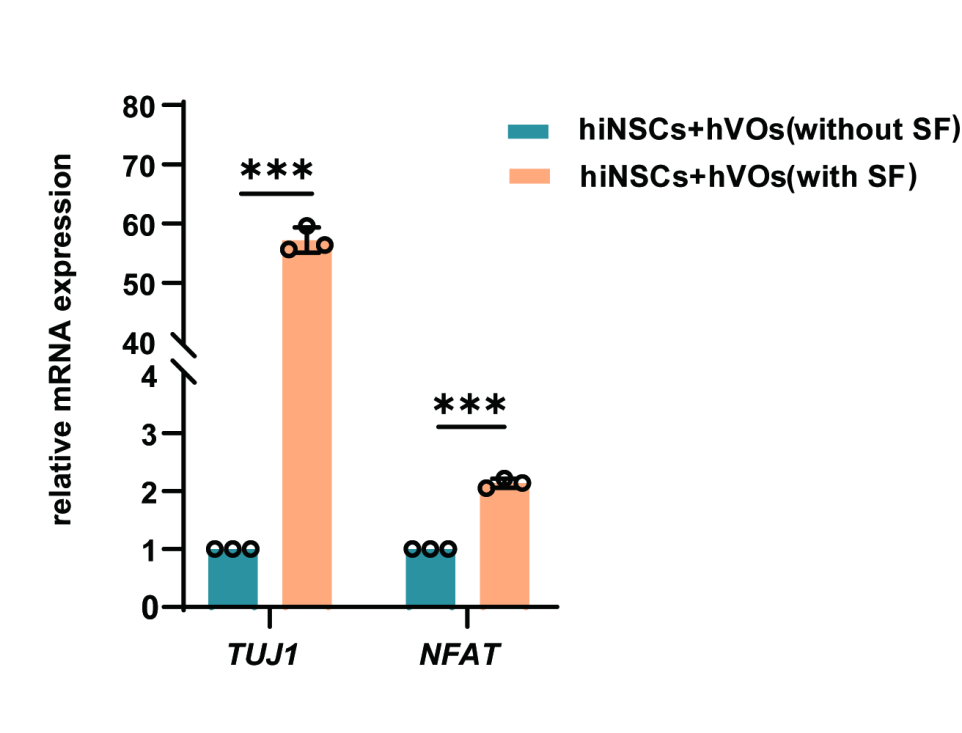


Relative mRNA expression of *TUJ1* and *NFAT* in hiNSCs co-cultured with hVOs in the presence or absence of oriented SF scaffolds.

RT-qPCR showing increased *TUJ1* and *NFAT* expression in the SF group, indicative of enhanced neuronal differentiation within the oriented SF scaffold (n = 3). ****P < 0.001.*

**Fig. S5**


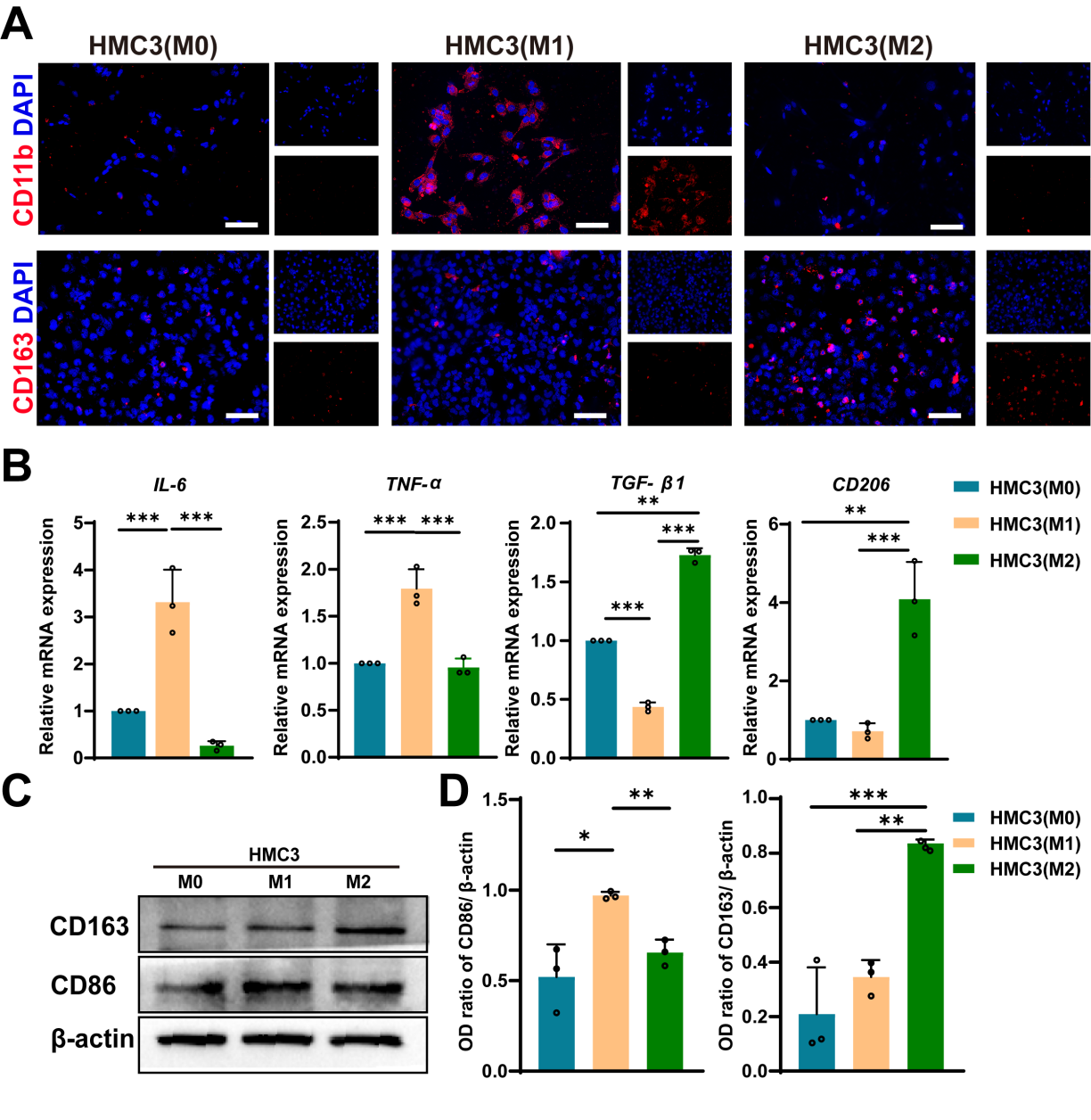


Characterization of distinct phenotypes of the human microglial cell line HMC3.

(A) Immunofluorescence staining showing predominant expression of CD11b in M1-polarized HMC3 cells, and the highest CD163 expression in M2-polarized cells, with reduced levels in control and M1 groups. Scale bar, 100 μm. (B) RT-qPCR analysis demonstrating elevated expression of *IL-6* and *TNF-α* in M1-polarized HMC3 cells, and increased expression of *TGF-β1* and *CD206* in M2-polarized cells (n=3). (C-D) Representative Western blot images and semi-quantitative analysis showing high CD86 expression in M1-polarized HMC3 cells and high CD163 expression in M2-polarized cells (n=3). **P* < 0.05, ***P* < 0.01, ****P* < 0.001.

**Fig. S6**


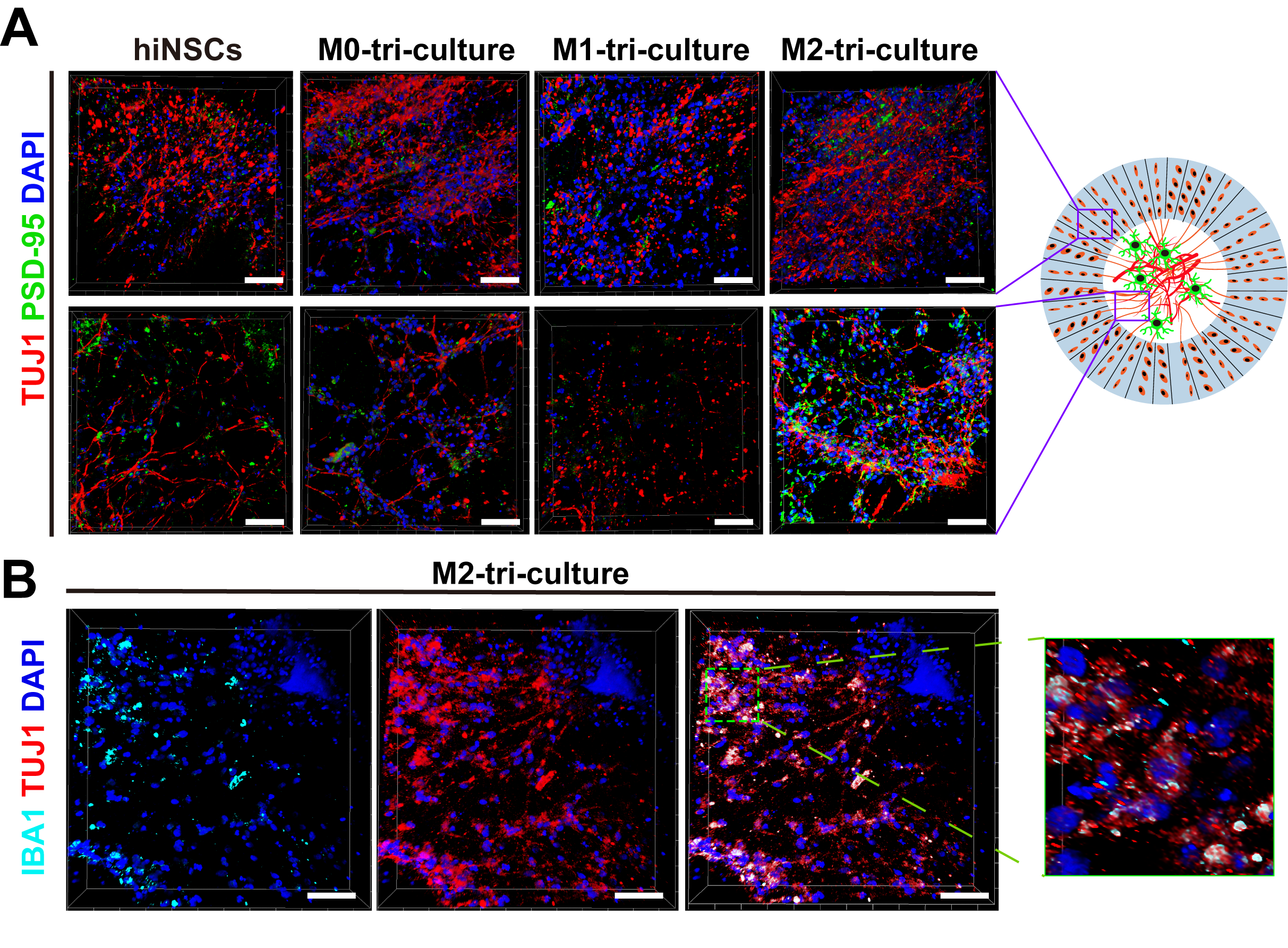


M2-polarized microglia promote neuronal maturation and spatial association with neurons in a tri-culture system.

(A) Representative immunofluorescence images showing enhanced axonal organization and synaptic density in the M2-tri-culture compared to M0 and M1 conditions. (B) Co-staining of IBA1 (cyan) and TUJ1 (red) in the M2 tri-culture model demonstrating close spatial proximity between M2-polarized microglia and neurons. All scale bar, 100 μm.

**Fig. S7**


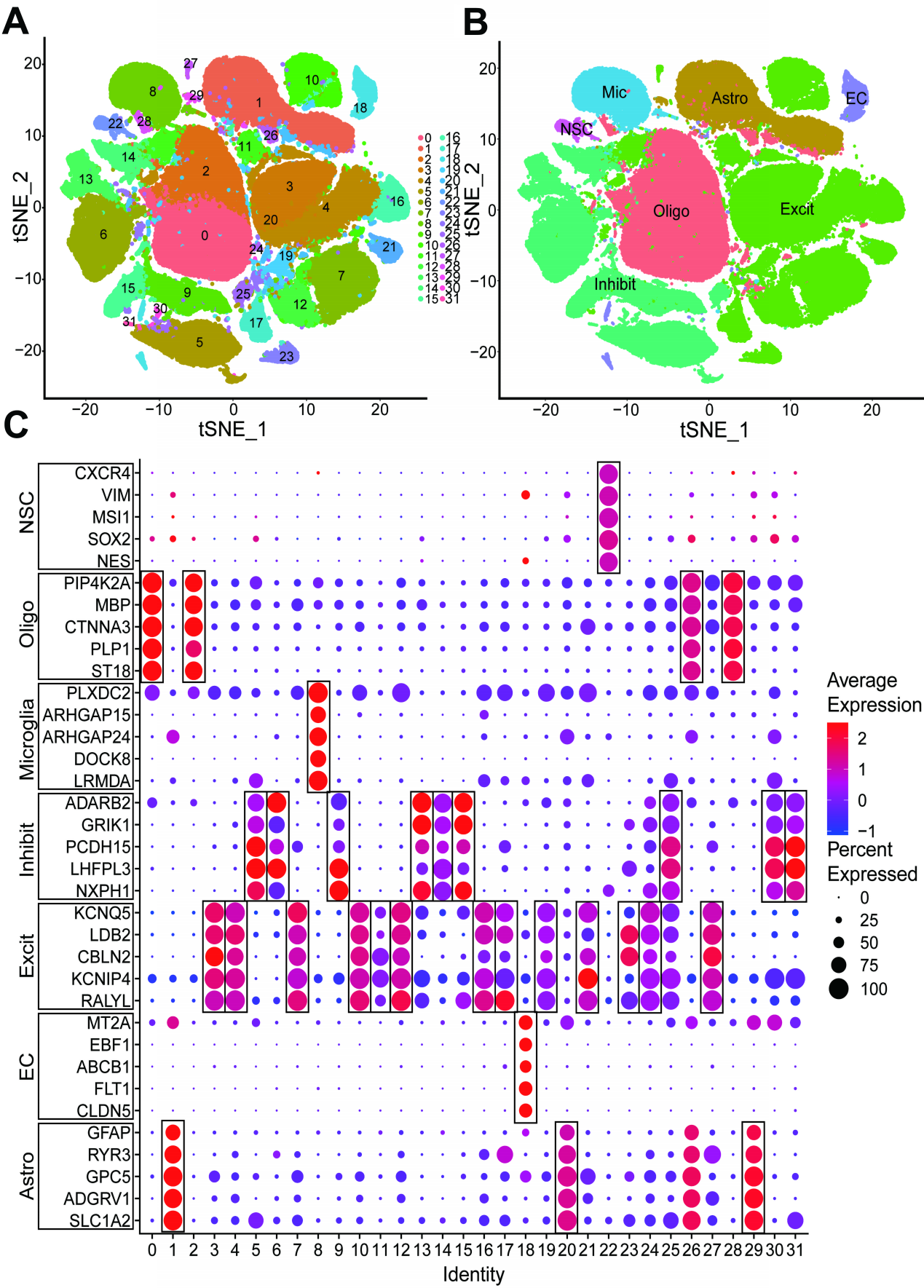
Single-cell clustering and cell-type annotation of the human prefrontal cortex.
(A) t-SNE plot showing 32 distinct cell clusters identified from the prefrontal cortex. (B) t-SNE plot displaying the annotation of 7 major cell types based on canonical marker genes. (C) Bubble plot illustrating representative marker gene expression across clusters. Dot size indicates the proportion of marker-positive cells, and color intensity represents average gene expression levels.

**Fig. S8**


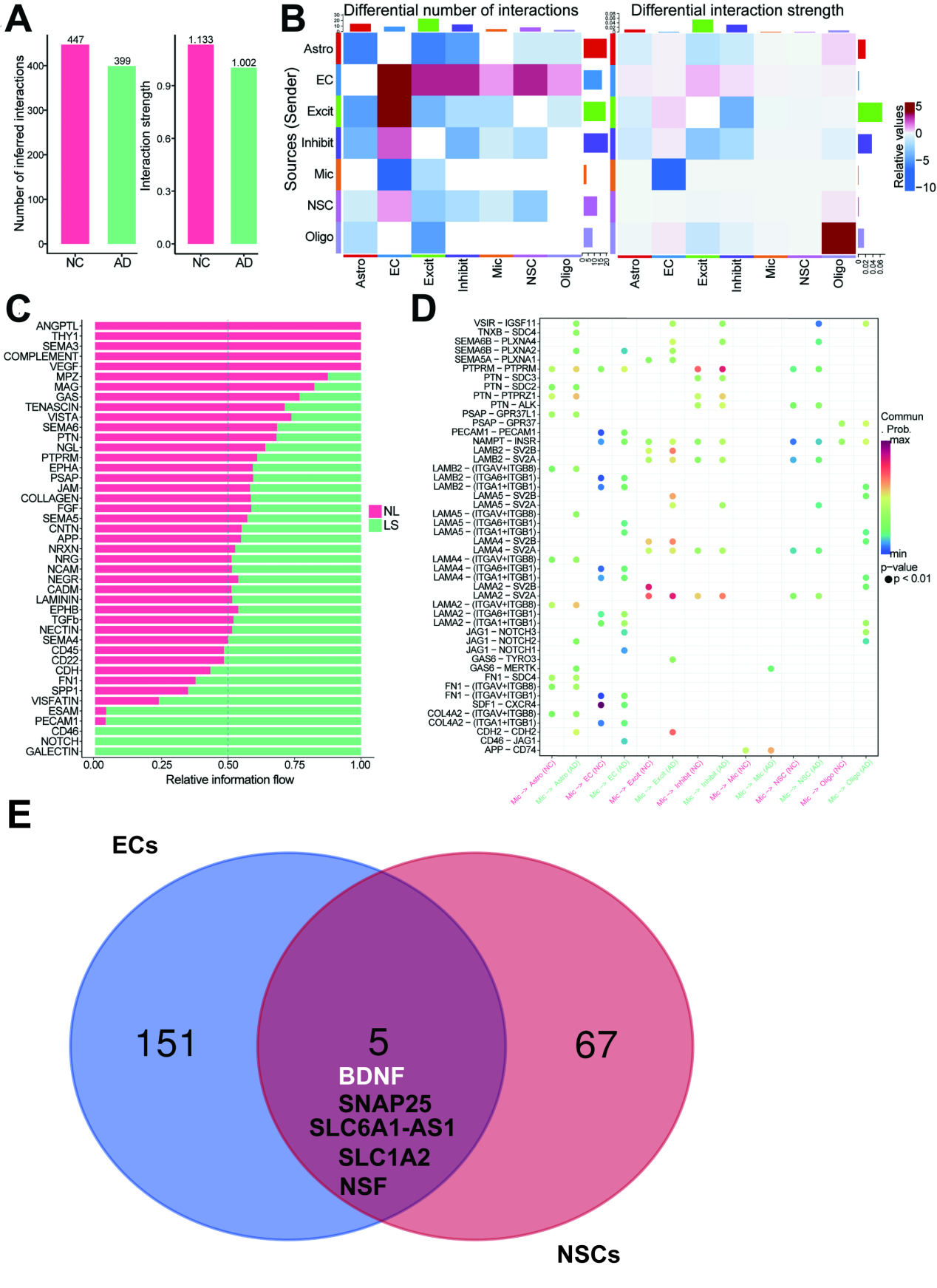


Altered cell-cell communication in the prefrontal cortex of cognitively normal individuals and Alzheimer's disease patients.

(A) Total number and overall strength of cell-cell interactions in the prefrontal cortex. (B) Heatmap showing changes in interaction number and strength between cell types in normal and AD samples. Red and blue indicate increased and decreased interactions, respectively. (C) Enrichment analysis of ligand-receptor pairs involved in cell-cell communication at the signaling pathway level, showing differentially enriched pathways between normal and AD samples for each cell type. (D) Overview of microglia-derived ligand-receptor interactions with other cell types in normal and AD samples, with the color scale indicating the mean expression of ligand-receptor pairs. Statistical significance assessed by permutation tests (*P* < 0.01). All analyses based on mRNA expression profiles and used to infer putative protein-level interactions. (E) Venn diagram of transcriptomic profiles in ECs and NSCs revealing shared expression of neurogenic factors.
