# Supplementary material for: Bioengineering an improved three-dimensional vascularized co-culture model for studying Neuron–Microglia interactions
Source: Bioact Mater. 2025 Sep 10;54:813–28. doi: 10.1016/j.bioactmat.2025.09.008 (PMC12628055; doi:10.1016/j.bioactmat.2025.09.008)
Supplement: Multimedia component 3 [file mmc3.docx]

**Supplementary table:**

**Table S1: Gene Primer sequences**

| **Gene name** | **Forward：5’-3’** | **Revers：5’-3’** |
| --- | --- | --- |
| *TUJ1* | GAACCCGGAACCATGGACAG | GACCCTTGGCCCAGTTGTTG |
| *CREST* | CAAGGCACCATCGGCAACTA | CTGCTGCATCATGGAGACTG |
| *NFAT* | GCTGGATAACAGTCGGATGTC | GCCTCTGCTTTGGATTTCGTT |
| *GAP-43* | GCTTCCGTGGACACATAAC | TGGTGCCTTCTCCCTTC |
| *SNAP-25* | ACCAGTTGGCTGATGAGTCG | CAAAGTCCTGATACCAGCATCTT |
| *PECAM1* | AGACGTGCAGTACACGGAAG | TTTCCACGGCATCAGGGAC |
| *VE-cadherin* | CGCAATAGACAAGGACATAACAC | GGTCAAACTGCCCATACTTG |
| *αSMA* | CTATGCCTCTGGACGCACAACT | CAGATCCAGACGCATGATGGCA |
| *PDGFRβ* | ATCAGCAGCAAGGCGAGC | CAGGTCAGAACGAAGGTGCT |
| *SYP* | CCGTGTTTGCCTTCCTCTACTC | CCCATGCCGATGAGCTAACT |
| *SOX2* | TACAGCATGTCCTACTCGCAG | GAGGAAGAGGTAACCACAGGG |
| *SDF-1* | CTGAAGAACAACAACAGACAA | GGCTCCACTTCAAATATATGAATT |
| *eNOS* | CCAGCTAGCCAAAGTCACCAT | GTCTCGGAGCCATACAGGATT |
| *CD206* | GCAGAAGGAGTAACCCACCC | TGGCAAATGAAGGCGTTTGG |
| *TNF-α* | CCAGGGACCTCTCTCTAATCA | TCAGCTTGAGGGTTTGCTAC |
| *IL-6* | GTAGTGAGGAACAAGCCAGAG | TGCATTTGCCGAAGAGCC |
| *TGF-β1* | ACCTGCCACAGATCCCCTAT | CTCCCGGCAAAAGGTAGGAG |
| *BDNF* | AGCCTCCTCTGCTCTTTCTGCTGGA | CTTTTGTCTATGCCCCTGCAGCCTT |
| *OCT4* | CCC CAG GGC CCC ATT TTG GTA CC | ACC TCA GTT TGA ATG CAT GGG AGA GC |
| *COL4A1* | TGCTGTTGAAAGGTGAAAGAG | CTTGGTGGCGAAGTCTCC |
| *GAPDH* | GCTTCCGTGGACACATAAC | TGGTGCCTTCTCCCTTC |
